# Nervous system development in the Pacific oyster, *Crassostrea gigas* (Mollusca: Bivalvia)

**DOI:** 10.1186/s12983-018-0259-8

**Published:** 2018-04-11

**Authors:** Olga V. Yurchenko, Olga I. Skiteva, Elena E. Voronezhskaya, Vyacheslav A. Dyachuk

**Affiliations:** 1 National Scientific Center of Marine Biology, Far Eastern Branch, Russian Academy of Sciences, Vladivostok, 690041 Russia; 2 Department of Physiology and Pharmacology, Karolinska Institutet, Stockholm, Sweden; 3 N.K. Koltzov Institute of Developmental Biology, Russian Academy of Sciences, Moscow, 119991 Russia; 4 Department of Pathology, Cell biology and Biochemistry, Central Tuberculosis Research Institute, Moscow, Russian Federation; 5 Far Eastern Federal University, Vladivostok, 690950 Russia; 6 Department of Neuroscience, Karolinska Institutet, Stockholm, Sweden

**Keywords:** Mollusca, Larvae, Neurogenesis, Evolution, Serotonin, FMRFamide, Acetylcholine

## Abstract

**Background:**

Bivalves comprise a large, highly diverse taxon of invertebrate species. Developmental studies of neurogenesis among species of Bivalvia are limited. Due to a lack of neurogenesis information, it is difficult to infer a ground pattern for Bivalvia. To provide more comprehensive morphogenetic data on bivalve molluscs and relationships among molluscan clades, we investigated neurogenesis in the Pacific oyster, *Crassostrea gigas*, from the appearance of the first sensory cells to the formation of the larval ganglionic nervous system by co-immunocytochemistry of the neuronal markers FMRFamide or 5-HT and vesicular acetylcholine transporter (VAChT).

**Results:**

Neurogenesis begins with the emergence of the apical serotonin-immunoreactive (5-HT-ir) sensory cells and paired sensory posttrochal dorsal and ventral FMRFamide-immunoreactive (FMRFamide-ir) cells at the early trochophore stage. Later, at the early veliger stage, the apical organ (AO) includes 5-HT-ir, FMRFamide-ir, and VAChT-ir cells. At the same stage, VAChT-ir cells appear in the posterior region of larvae and send axons towards the AO. Thus, FMRFamide-ir neurites and VAChT-ir processes form scaffolds for longitudinal neurite bundles develop into the paired ventral nerve cords (VNC). Later-appearing axons from the AO/CG neurons join the neurite bundles comprising the VNC. All larval ganglia appear along the VNC as paired or fused (epiathroid) clusters in late veliger and pediveliger larvae. We observed the transformation of the AO into the cerebral ganglia, which abundantly innervated the velum, and the transformation of ventral neurons into the pedal ganglia, innervating the foot, gills, and anterior adductor muscle. The visceral ganglia appear last in the pediveliger oyster and innervate the visceral mass and posterior adductor of premetamorphic larvae. In addition, a local FMRFamide-ir network was detected in the digestive system of pediveliger larvae. We identified VAChT-ir nervous elements in oyster larvae, which have not been observed previously in molluscs. Finally, we performed a morphology-based comparative analysis of neuronal structures among bivalve, conchiferan, and aculiferan species.

**Conclusions:**

We described the development of the nervous system during the larval development in *Crassostrea gigas*. These data greatly advance the currently limited understanding of neurodevelopment in bivalves and mollusks, which has hampered the generation of a ground pattern reconstruction of the last common ancestor of Mollusca. Our morphological data support phylogenomic data indicating a closer Bivalvia-Gastropoda sister group relationship than the Bivalvia-Scaphopoda (Diasoma) group relationship.

**Electronic supplementary material:**

The online version of this article (10.1186/s12983-018-0259-8) contains supplementary material, which is available to authorized users.

## Background

Bivalves comprise a large taxon of invertebrates with a high degree of variation in development (planktotrophic and lecithotrophic larvae), life style (sedentary or sessile), and morphology (adaptive reduction of inner organs), as well as ecological diversity (marine, freshwater, tropics and Arctic waters). Recent transcriptomic characterizations of bivalves using RNA-seq data has arranged them into five clades (Protobranchia, Pteriomorpha, Palaeoheterodonta, Archiheterodonta, and Euheterodonta) [1]. Most bivalves exhibit a biphasic life cycle that includes actively swimming plankton larval stages and sedentary benthic adult animals [2]. Neurogenesis in this animal group has been explored to a limited extent by zoologists and morphologists, especially in the larval stage, with only a few detailed descriptions in the literature [3–6].

The subject of the present study is the Pacific oyster, *Crassostrea gigas* (Pteriomorphia: Ostreida, Thunberg, 1793), which is one of the commonly found molluscs in the world [7]. The nervous system of the adult oyster *Crassostrea virginica* consists of central and peripheral branches. The central nervous system comprises paired cerebral ganglia lying symmetrically on both sides of the molluscan body and a huge visceral ganglion in which the right and left components are fused into a single organ [8]. Cerebral ganglia are located in the esophageal region and are connected by a U-shaped commissure. The visceral ganglia, which are the major constituent of the oyster nervous system, are located in the most caudal part of the body and are connected to the cerebral ganglia via long cerebro-visceral. Cerebro-pedal and pedal-visceral connectives form paired ventral nerve cords (VNC) described in molluscs and billaterians. In the adult oyster, the pedal ganglia and the cerebro-pedal connections are reduced due to the loss of the foot after metamorphosis [8]. The peripheral nervous system includes numerous nerves that extend from the ganglia that innervate the mantle edge, gills, and other parts of the body.

Data on neurodevelopment in larval bivalves are sparse, particularly for oysters [3, 9, 10], and in many cases, such investigations have been restricted to studies of a single neuroactive substance [6], a strongly modified mode of development [11], or late developmental stages [4, 12, 13]. Further, the different methodological approaches used make it difficult to perform a comparative analysis of the neurodevelopment of various groups or species. For example, histological data have been used to provide a detailed description of neurogenesis in the oyster *Crassostrea virginica* [14], whereas only immunochemical data are available for the Pteriomorpha (*Mytillus trossulus* and *Mytillus edulis*) [4, 5] and Imparidentia (*Dreissena polymorpha, Spisula solidissima*) [15, 16].

Knowledge of the emergence, development, and organization of the larval oyster nervous system is essential for understanding the role of the nervous system in larval survival and adaptation. Moreover, developmental ontogenetic data are necessary to demonstrate the degree to which neurogenesis reflects ancestral traits. Therefore, the search for the evolutionary origin and phylogenetic sister-groups of bivalves, as well as Mollusca in general, is ongoing. Based on segmentation there are known to be numerous synapomorphies that share neuro-developmental characteristics (e.g., structure of the larval apical organ and nerve cords) in larval entoprocts, aplacophorans, and polyplacophorans as well as and similarities in bivalve and scaphopod neurogenesis. Considering these observations along with anatomical, embryological and paleontological data, several phylogenetic hypotheses and concepts have been suggested, including the diasoma concept [17], the annelid-mollusk hypothesis [18, 19], and the tetraneuralia concept [20]. These competing ideas are difficult to confirm and reconcile due to the lack of information provided by fossil records and insufficient morphological data related to neurogenesis in mollusks, particularly bivalve species.

The aim of the present study was to elucidate details about the main events in larval bivalve neurogenesis and to discuss the possible defining characteristics of the last common ancestor of Bivalvia and Conchifera. Therefore, we examined neuronal development in *Crassostrea gigas* larvae from the appearance of the first sensory cells and their neurite pathways to the formation of the larval nervous system, including innervation of inner organs in oyster pediveligers. Characterizations were made with the neuronal markers serotonin (5-HT) and Phe-Met-Arg-Phe amide (FMRFamide). In addition, we examined immunoreactivity against vesicular acetylcholine transporter (VAChT) and choline acetyltransferase (ChAT) as presumptive markers of acetylcholine-containing neurons.

## Methods

### Animals

Mature oysters (*Crassostrea gigas*) were collected from the estuary at the “Vostok” marine biological station of the National Scientific Center of Marine Biology of the Far East Branch of the Russian Academy of Sciences (NSCMB FEB RAS) (Vostok Bay and Peter the Great Bay of the Sea of Japan 42°48′34,9″ W, 142°54′.18,9″ L, 10 m depth), during July 2014 and July 2015. Standard techniques [21], with some modifications, were used to obtain larval cultures. Briefly, spermatozoa and mature oocytes were obtained by gonad stripping of adult oysters. Larval cultures were kept at 20 °C in 5-L beakers containing filtered sea water with constant agitation of the water column by an air jet directed at the water surface; the water was changed every 3 days. Starting from 36 h post-fertilization (hpf), larvae were fed microalgae (*Isochrysis galbana* and *Chaetoceros muelleri*; 100,000 cells/mL), and an additional mixture of *Dunaliella salina* and *Phaeodactylum tricornutum* (50,000 cells/mL) was added to the larval rations after 10 days post-fertilization (dpf). The subsequent stages of larval development were examined under a Zeiss Axio Imager Z2 light microscope (Carl Zeiss, Jena, Germany) equipped with a digital camera (Axio Cam Hrc, Carl Zeiss) using bright field or differential interference contrast techniques (Far East Center of Electron Microscopy, NSCMB, FEB RAS).

For morphological studies, oyster larvae were fixed at the following stages: blastula (12 hpf); early, middle, and late trochophore stages (20, 24, and 28 hpf, respectively); and the early (D-) (36–52 hpf), middle (92–96 hpf), and late veliger stages (5–9, and 15 dpf), as well as the pediveliger stage (28–35 dpf).

### Western blotting

For western blot analysis, samples of adult oyster muscle, mantle, and gills were taken, and samples of the cervical and thoracic parts of mouse spinal cord were used as a positive control (mice were obtained from the Department of Physiology and Pharmacology, Karolinska Institute). Lysates were prepared by sonication in 1% sodium dodecyl sulfate (SDS), and homogenates were incubated at 98 °C for 10 min and centrifuged for 5 min at 13,000 *g*. The protein concentrations in the samples were estimated using a Pierce™ BCA Protein Assay Kit (Thermo Fisher Scientific, Waltham, MA, USA), after which Laemmli sample buffer was added to the samples. Polyacrylamide gels (9%) were loaded with samples (90 μg of protein/well), and electrophoresis was conducted at 90 V in 1 × Tris/glycine/SDS running buffer. Proteins were transferred to a nitrocellulose membrane (75 min at 80 V in transfer buffer containing 0.3% Tris, 1.44% glycine, and 30% methanol). Nonspecific binding was blocked by a 1-h incubation of the membrane in blocking buffer (TBS-T, 5% powdered milk), and membranes were then incubated overnight at 4 °C in blocking buffer with goat anti-VAChT (1:500) or anti-ChAT (1:500) antibodies (Table 1). Subsequently, membranes were washed six times in TBS-T for 5 min, after which membranes were incubated for 2 h in blocking buffer with horseradish peroxidase (HRP)-linked anti-goat IgG (1:1000; Table 2) at room temperature and then washed six times for 5 min. Immunoreactivity was detected using Clarity™ Western ECL Substrate (BioRad, Hercules, CA, USA).

**Table 1:** Primary and secondary antibodies used in the study

Antibody	Host species	Source, Cat. #	Dilution
Acetylated α-tubulin	Mouse monoclonal	Abcam, 6-11B-1	1:2000
FMRFamide	Rabbit polyclonal	Immunostar, 20091	1:2000
5-HT	Rabbit polyclonal	Immunostar, 20080	1:2000
ChAT (choline acetyltransferase)	Goat polyclonal	Millipore, AB144P	1:500
VAChT (vesicular acetylcholine transporter)	Goat polyclonal	Millipore, ABN100	1:500
AlexaFluor 555 anti-goat	Donkey	Life Technologies, A21432	1:1000
AlexaFluor 488 anti-rabbit	Donkey	Life Technologies, A21206	1:1000
AlexaFluor 555 anti-rabbit	Donkey	Life Technologies, A31572	1:1000
AlexaFluor 647 anti-mouse	Donkey	Life Technologies, A31571	1:1000
AlexaFluor 555 anti-mouse	Donkey	Life Technologies, A31570	1:1000

**Table 2:** Morphological and Anatomical Features of *Crassostrea gigas* Larval Development Stages

Stage^a^	Time of development^b^	Morphological features
Early trochophore	20 hpf	Spheroidal form, slightly conical at the basal region with two obvious invaginations (shell gland and presumptive mouth opening) and prototroch (Fig. 1a). Long cilia form an apical tuft that is present at the apical pole.
Middle trochophore	24 hpf	Spheroidal form with one invagination (mouth). Shell gland everted (Fig. 1b).
Late trochophore	28 hpf	Spheroidal form with well-developed prototroch and telotroch. Shell gland begins to secrete a shell (Fig. 1c). Locomotory organ—prototroch.
Early veliger (D-hinge-stage veliger)	36 hpf—72 hpf	Two D-shaped shells surround the larval body. Digestive tract is complete and consists of mouth, esophagus, stomach, intestine, and anus. Elements of the muscular system (retractors) are well developed. Locomotory organ—velum (Fig. 1d).
Middle veliger	92 hpf-96 hpf	Shells increase in size and are slightly elongated. Retractors and two adductors are well developed (Fig. 1e).
Late veliger	after 5 dpf	Right and left shells first demonstrate asymmetry (Fig. 1f). Velum is markedly larger than in the previous stage.
Pediveliger (umbo stage)	after 28 dpf	Umbo is distinguished as highest and most prominent part of each valve of the larval shell. Velum is markedly larger than at the previous stage.

*hpf* hours post fertilization, *dpf* days after fertilization

^a^Oyster developmental stages were recognized by external morphological characteristics rather than based on exact determination of time after fertilization

^b^The data are for larval development at a temperature of + 20 °C and salinity of 28‰

### Immunohistochemistry

Prior to fixation, larvae were relaxed in a solution of 7% MgCI2 in 0.1 M phosphate-buffered saline (PBS, pH 7.4). After relaxation, larvae were fixed in 4% paraformaldehyde in 1 × PBS for 5 h at 4 °C and rinsed for 15 min in 0.1 M PBS with 0.03% NaN_3_ three times. Immediately thereafter, larvae at veliger stages were treated with 0.1 M EDTA for 1 h at room temperature and washed in PBS three times to decalcify their shells. Larvae were permeabilized in 1× PBS with 0.03% NaN3 and 1% Triton X-100 (PBST) for 15 min at room temperature and then incubated overnight in blocking solution (10% normal goat serum, 1% bovine serum albumin [BSA] in PBST) at 4 °C to eliminate non-specific binding sites. The larvae were subsequently incubated with primary antibodies (Table 1) at a final dilution of 1:2000 in blocking solution for 3–5 days at 4 °C using a monoclonal antibody against acetylated α-tubulin (Sigma, St Louis, MO, USA) or polyclonal antibodies against FMRFamide or 5-HT (Immunostar, Hudson, WI, USA), and VAChT or ChAT (Millipore, Darmstadt, Germany). After triple rinsing in PBST, the larvae were incubated overnight at 4 °C with secondary antibodies (Table 2), including AlexaFluor 488 goat anti-rabbit IgG (GAR), AlexaFluor 546 goat anti-mouse IgG (GAM), and AlexaFluor 633 GAM (Molecular Probes, Waltham, MA, USA) that had been diluted to 1:1000 in 1× PBS. To detect the larval muscle system, AlexaFluor 555-labelled phalloidin (Molecular Probes, diluted to 1:1000) was used.

For co-localization analysis of FMRFamide and 5-HT, larvae were first incubated with the FMRFamide antibody followed by the AlexaFluor 546 GAR secondary antibody. The larvae were then incubated with a mixture of the anti-5-HT and anti-acetylated α-tubulin antibodies followed by incubation with the AlexaFluor 488 GAR and AlexaFluor 633 GAM antibodies. Each incubation was followed by three 15-min washes in PBST.

Finally, larvae were immersed in glycerol with 2.5% DAPCO antifade (Sigma). At least 50 embryos were examined at each stage using each of the staining protocols, and negative controls were obtained by omitting the primary antibodies to demonstrate absence of specific staining with secondary antibodies. All samples were viewed under Leica SPE (Wetzlar, Germany) or Zeiss LSM 810 confocal laser scanning microscopes. Images were processed with Illustrator CS6 (Karolinska Institute, Solna, Sweden), and 3D reconstructions were created using Imaris 7.0 (Karolinska Institute). Adult oyster tissues were prepared for immunohistochemistry using a previously described protocol [22].

### Primary antibodies

To describe larval neurogenesis in the present study, we used antibodies (Table 1) against rabbit polyclonal FMRFamide (Cardio-excitatory Peptide, Immunostar) and rabbit polyclonal serotonin (5-HT, Immunostar). FMRFamide and serotonin antibodies from Immunostar were quality control tested using standard immunohistochemical methods. The antiserum demonstrated strongly positive labeling of rat hypothalamus and spinal cord in indirect immunofluorescence assays, and the specificity of FMRFamide and serotonin antibodies has repeatedly been confirmed in bivalve species using immunohistochemistry [5, 15, 23, 24]. FMRFamide and serotonin antibodies are widely used markers for detecting neuronal elements in different invertebrate taxa. Details of the primary and secondary antibodies used are presented in Table 1.

Cholinergic neuronal elements were detected using goat polyclonal choline acetyltransferase antibody (ChAT, Millipore) and goat polyclonal vesicular acetylcholine transporter antibody (VAChT, Millipore). As this antibody has not been previously used in bivalve tissues, we first tested whether the antibody specifically recognized *Crassostrea* ChAT and VAChT in western blotting and immunohistochemistry of oyster adult tissues and larval materials. As a positive control for ChAT and VAChT labeling, we used total protein extracts from mouse spinal cord. VAChT, but not the ChAT antibody, recognized a band of the expected molecular weight in adult tissues and in whole-mount immunostaining oyster preparations.

All neuronal antibodies used here were combined with antibodies against monoclonal acetylated tubulin (Abcam, Cambridge, MA, USA). This antibody is widely used to detect microtubules in the ciliary systems and nervous system elements of invertebrate taxa [25].

## Results

### Morphology of the main developmental stages of *Crassostrea gigas*

**Fig. 1:**
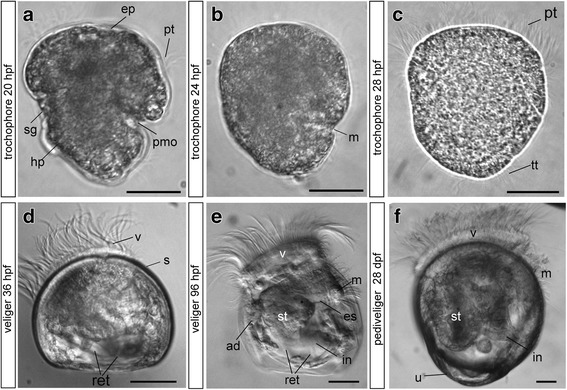
Stages of *Crassostrea gigas* larval development. Light microscopy images. Early (**a**), middle (**b**), and late (**c**) trochophores and early (D-hinge, **d**), middle (**e**), and pedi-umbo-stage (**f**) veligers. Abbreviations: ad - anterior adductor, ep – episphere, es – esophagus, hp. – hyposphere, in – intestine, m – mouth, pmo – presumptive mouth opening, pt. – prototroch, ret. – retractors, s – shell, sg – shell gland, st – stomach, tt – telotroch, u – umbo, v – velum. Scale bars = 20 μm

The early trochophore (20 h post fertilization, hpf) has a spheroidal form and is slightly conical in the basal region, with two obvious invaginations (the shell gland and presumptive mouth opening). Cilia form the locomotory organ, i.e., the prototroch, that divides the body into the upper episphere (or pretrochal region) and the lower hyposphere (or posttrochal region) (Fig. 1a). Additional long cilia form a tuft that is present at the apical pole of the larva. The middle trochophore (24 hpf) has a similar form, but only one invagination is visible (the mouth) on the larval body (Fig. 1b), whereas the shell gland is everted. At the late trochophore stage (28 hpf), the shell gland secretes a shell. The prototroch and telotroch are well developed (Fig. 1c, Additional file 1: Figure S1), and the early veliger or D-hinge (36 hpf) stages demonstrate a D-shaped shell form (Fig. 1d). The locomotory organ of the veliger stages is the velum, which derives from the prototroch. From this stage onward, the larvae have a fully developed digestive tract including a mouth, esophagus, stomach, intestine, and anus (Additional file 1: Figure S1). The shells of the later stages (middle and late veliger) differ from that of the early stage in that they are higher and have a more rounded shape (Fig. 1e). At the pediveliger stage, the hinge develops two well-distinguished bulges or umbones; the one on the left side is larger than its opposite structure (Fig. 1f). Some features of the developmental stages of the studied oyster are summarized in Table 2.

### FMRFamide-immunoreactive structures

**Fig. 2:**
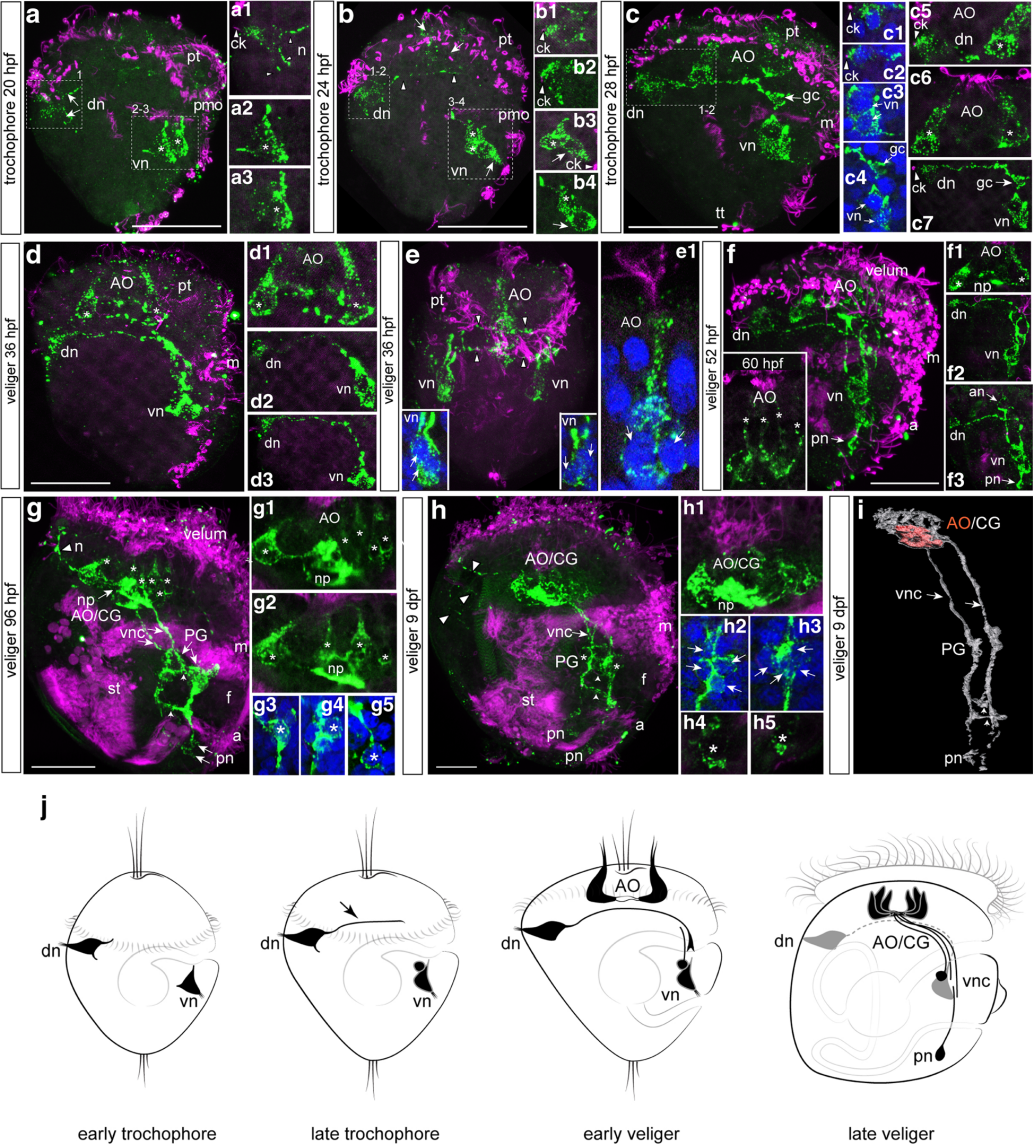
FMRFamide-immunoreactivity (FMRFa-ir) in the trochophore and veliger larvae of *C. gigas.* Green—FMRFa-ir; magenta—cilia, acetylated tubulin immunoreactivity; blue—nuclei, DAPI. The apical pole is always upward; the ventral side is on the right. **a** Trochophore at 20 hpf. Dorsal (arrowheads) and ventral (asterisks) groups of early neurons located posttrochally. **a1** Higher magnification of right dorsal neuron (dn); arrowheads indicate a ciliated dendritic knob (ck) and basal neurites (n). **a2** and **a3** Right and left ventral neurons (vn, asterisks), respectively. **b** Trochophore at 24 hpf. Faint dotted immunoreactivity appears at the apical region (*arrows*). A dorsal cell sends an axon towards the ventral side (*arrowheads*). Three ventral cells indicated by one *arrow* (flask-shaped cells) and asterisks (round cells). **b1 and b2** Higher magnification of the right and left dorsal neurons (dn), arrowheads indicate the ciliated dendritic knob (ck) of each cell. **b3** and **b4** Higher magnification of right and left ventral groups (vn); each ventral group contains two flask-shaped cells (*arrows*) and two round cells (asterisks). **c** Trochophore at 28 hpf. Two flask-shaped cells appear in the apical organ (AO). Growth cone (*arrows*, gc) of the right dorsal cell (dn), where a growing axon reaches the ipsilateral ventral cell (vn); only the right sides of the larvae are shown. **c1-c4** Right and left dorsal and ventral groups of neurons with DAPI. **c5** and **c6** Two flask-shaped neurons of the AO at two different magnifications (asterisks). **c7** Higher magnification of a growing axon of the right dorsal cell; arrowhead indicates a ciliated dendritic knob, and arrows indicate a growth cone of the dorsal neuron. **d** D-hinge veliger stage at 36 hpf. The cells of the AO are located at the top (asterisks); no connections exist between the AO and other early cells. **d1** Two flask-shaped apical neurons (asterisks) and their basal neurites. **d2** and **d3** Long axons of both the right and left dorsal cells reach the ipsilateral groups of the ventral cells. **e** Ventral view of the veliger at 36 hpf. Arrowheads indicate projections from dorsal and apical cells towards the ventral neurons. **insets** Higher magnification of the left and right ventral groups. **e1** Higher magnification of the apical neurons demonstrating long cilia at the end of their dendrite (*arrows*). **f** The veliger stage at 52 hpf and 60 hpf (insert). Dorsal (dn) and ventral (vn) cells stain in a punctate pattern and their axons form an anlagen of the ventral nerve cord. Small immunopositive neurons appear posteriorly (posterior neurons, *arrow*, pn). Insert: Four flask-shaped apical neurons (asterisks) of AO.**f1** Higher magnification of the AO (asterisk). **f2** Long axon of a dorsal neuron (dn) to the ventral neuron (vn). **f3** Neurites extending from the AO (arrow, an) follow the path established by pioneer axons of the dorsal neurons. **g** The veliger stage at 96 hpf. AO/CG consist of six flask-shaped cells (asterisks) and basal processes of AO/CG neurons organize into a compact central neuropil (*arrow*, np). A single neurite (n, *arrowhead*) extends from the neuropil of the AO/CG toward the velum. Paired ventral cords (vnc) with the interconnecting commissures (*short arrows*) are clearly visible. The anlagen of the pedal ganglia (PG) appear along each ventral cord in the region of the developing foot. **g1** Flask-shaped neurons (asterisks) with neuropil forming (Z12). **g2** Flask-shaped neurons (asterisks) with neuropil forming (Z14). **g3** and **g4** Neurons within the PG under high magnification (asterisks). **g5** Posterior neurons under high magnification (asterisk). **h** The late veliger stage at 9 dpf. Neurites extend from the AO/CG to the dorsal side and dorsal edge of the velum (*arrowheads*). The anlagen of the PG are located along the ventral cord (vnc). Paired ventral cords are connected by two commissures (*short arrows*). **h1** Compact neuropil in the center of the AO/CG. **h2** and **h3** Right and left PG anlagen; *arrows* point to the neuronal nuclei. **h4** and **h5** Higher magnification of the right and left posterior neurons; the *asterisk* marks the cell nucleus. **i** 3D reconstruction of the AO/CG complex, paired ventral cords and PG anlagen of 9-dpf late-veliger larvae. **j** Summary diagram of the ontogeny of the FMRFa-ir-containing structures in *C. gigas.* Only one lateral side is shown. Arrowhead indicates an axon of a dorsal cell in the late trochophore. Additional abbreviations: a – anus, f – foot, m – mouth, pmo – presumptive mouth opening, pt – prototroch, st – stomach. Scale bars =20 μm

No FMRFamide immunoreactivity is observed in the blastula and early trochophore stages, before 20 hpf (data not shown). The first FMRFamide-immunoreactive (FMRFa-ir) elements appear as two discrete groups of neurons at the early trochophore stage (20 hpf), and the first pair of FMRFa-ir cells is located posttrochally in the dorsal region (Fig. 2a and j). The two cells lay symmetrically and closely adjacent to each other, caudally to the prototroch. Two neurites extend from each cell. A thick short dendrite passes through the epithelium and bears a short bunch of cilia; a thin short axon extends caudally; and a thin long axon extends anteriorly (Fig. 2a1). The second pair of FMRFa-ir cells also lay posttrochally but caudally to the mouth in the ventral region (Fig. 2a). Each triangular-shaped ciliated cell is located symmetrically on the right and left sides of the larvae (Fig. 2a2 and a3).

At the middle trochophore stage (24 hpf), faint dotted immunoreactivity appears at the apical region (Fig. 2b), and the number and position of the dorsal cells do not change (Fig. 2b1 and b2). An anteriorly running axon projects further into the ventral side from each dorsal sensory FMRFa-ir cell, following the curvature of the anterior hemisphere, and a total of four cells (2 flask-shape with cilia and two round cells) are now detected in the ventral group (Fig. 2b3-b4 and j).

In the late trochophore stage (28 hpf), FMRFamide-ir becomes more prominent within the bodies and thick apical dendrites of the anterior apical cells, forming the apical organ (AO) (Fig. 2c, c5, c6 and j). At this stage, the positions of the paired sensory dorsal (Fig. 2c1 and c2) and ventral cells (Fig. 2c3, 2c4) do not change. Long axons of dorsal cells run underneath the flask-shaped anterior cells (Fig. 2c), and a brightly visible growth cone of the right dorsal cell axon reaches the ipsilateral ventral cell group (Fig. 2c7 and j).

By the early D-hinge veliger stage (36 hpf), the cell bodies of the apical cells and their basal neurites comprise a compact AO that has no connections with dorsal or ventral cells and their processes (Fig. 2d, d1, and e); the dendrites of the apical cells bear long cilia (Fig. 2e1). At this stage, two cells are visible in each of the ventral groups (Fig. 2d2, d3, and E insets), and long neurites of both the right and left dorsal cells reach the ipsilateral groups of the ventral cells (Fig. 2d2 and d3). Thus, two parallel (left and right) processes of dorsal and ventral FMRFa-ir cells form the anlagen of the paired ventral nerve cords (Fig. 2d, d2, d3 and j).

In the early veliger stage (52 hpf), FMRFa-ir labeling within the cell bodies of both the early dorsal and ventral cells becomes punctate (Fig. 2f), while the long axons of the dorsal neurons are still visible. Processes emanating from the AO run along this connection (Fig. 2f1-f3), and solitary small immunopositive neurons appear posteriorly (Fig. 2f-f3 and j). Later, (60 hpf) four flask-shaped apical neurons (asterisks) of AO were detected (Fig. 2f, insert).

At the middle veliger stage (96 hpf), the cell bodies of the dorsal groups of early-appearing cells are no longer detected (Fig. 2g), and there is also no sign of the dendritic knob of the dorsal cells on the dorsal side of the larval body. The basal processes of the AO neurons are organized into a compact central neuropil, and six flask-shaped cells form the complex spatial structure, i.e., the apical/cerebral ganglion (AO/CG) (Fig. 2g1, 2g2 and j). A single neurite extends from the neuropil of the AO/CG toward the velum, and two commissures connect the right and left ventral cords (Fig. 2g). The anlagen of the pedal ganglia (PG) appear in the region of the developing foot and along each ventral cord (Fig. 2g3 and g4) enveloping the esophagus. Solitary posterior cells are located at the caudal end of each ventral cord (Fig. 2g5).

At the late veliger stage (9 days post fertilization, dpf), FMRFa-ir cells within the apical/cerebral ganglion become more compact (Fig. 2h and h1), and neurites extending from the AO/CG run to the dorsal side of the velum. The anlagen of the PG (Fig. 2h2 and h3) are located adjacent to the paired ventral cord, which is connected by two commissures (Fig. 2h) and terminates with posterior neurons (Fig. 2h4 and h5). Thus, the 9-dpf late veliger stage possesses the AO/CG complex, paired ventral cords with interconnecting commissures, paired pedal ganglia, and posterior neurons (Fig. 2i). We further characterized the neuroanatomy of the FMRFamide system and innervation patterns in the pediveliger, as is detailed later in the paper (Fig. 6).

### 5-HT-immunoreactive structures

**Fig. 3:**
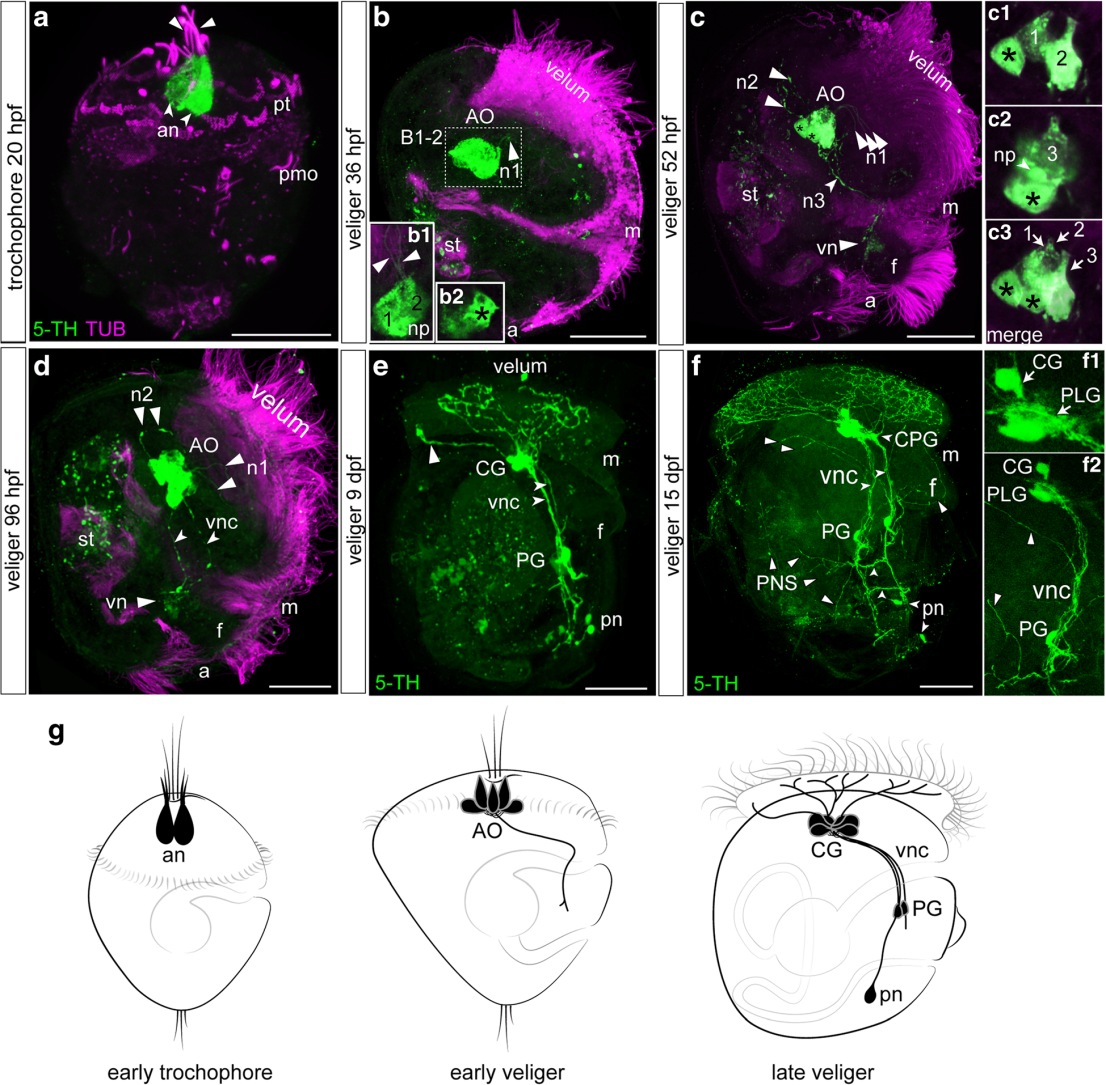
Serotonin immunoreactivity (5-HT-ir) in the trochophore and veliger larvae of *Crassostrea gigas***.** Green—5-HT-ir; magenta—cilia, acetylated tubulin immunoreactivity. The apical pole is always upward; the ventral side is on the right. **a** The early trochophore at 20 hpf. The first two flask-shaped neurons (*arrows*, an) are located in the anterior extreme of the larval body. Each cell has a ciliated short dendrite (*arrowheads*). **b** D-hinge veliger at 36 hpf. 5-HT-ir cells comprise a compact apical organ (AO) and comprise flask-shaped and round cells each with thin neurites (*arrowheads*, n1) running to the velum. **b1** two flask-shaped cells with thin neurites and neuropil (np). **b2** single round cells (black asterisk). **c** The veliger stage at 52 hpf. Cells of the AO extend three long neurites to the velum (*arrowheads*, n1), anterior−dorsal (*arrowheads*, n2), and posterior−ventral (*arrowheads*, n3). Acetylated tubulin-ir reveals the digestive system and green autofluorescent particles are visible in the stomach (st) in b, c, d, e. **insets:** High magnification of the AO cell composition. **c1** two flask-shaped and one round cells (black asterisk) (Z5), **c2** third flask-shaped cells and second round cell (black asterisk), and neuropil (*arrows*, np) **c3** merge picture. There are five cells: three flask-shaped cells and two round cells (black asterisk); **d** The middle veliger stage at 96 hpf. Two posterior−ventral neurites from cells of the AO run in parallel along the ventral part of the larval body organizing the ventral nerve cords (vnc). **e** The veliger at 9 dpf. Cerebral ganglia (CG) are located at the top, and pedal ganglia (PG) are detected in the middle of the ventral nerve cord. Small immunoreactive posterior neurons (pn) are located near the caudal end of each ventral cord. Neurites extending from the CG have multiple branches in the velum region. Note the solitary branch extending from the apical part of the ganglion (*arrowhead*) towards the dorsal edge of the velum. **f** The veliger at 15 dpf. Note the paired ventral nerve cords with two commissures (*short arrows*). Cerebral ganglia located on top of the ventral cords. The thickening of the upper portion of each ventral cord represents the anlagen of the pleural ganglia (PLG) and together with the CG they form a fused CG/PLG complex indicated as CPG. Right and left PG are located along the respective ventral cords at the middle of the foot. Posterior neurons are located caudally, and each sends an axon to the ipsilateral ventral cord. The velum is richly innervated by fibers extending from the CPG. Thin neurites extend to the foot, and digestive organs (mark as peripheral nerve system, PNS) extend from the PG region (*arrowheads*). **insets: f1** High magnification of the CG/PLG region; **f1** focus on CG and PLG parts of CPG, PG, ventral cord, and peripheral innervation. **g** Summary diagram of the ontogeny of the 5-HT-ir-containing structures in *C. gigas*. Only one lateral side is shown. Additional abbreviations: f – foot, m – mouth, pmo – presumptive mouth opening, pt. – prototroch, st – stomach. Scale bars =20 μm

No 5-HT-immunoreactive elements are observed at the blastula and early trochophore stages before 20 hpf (data not shown). The earliest two 5-HT immunoreactive (5-HT-ir) cells are detected at the anterior extreme of the larval body at the early trochophore stage (20 hpf). Two flask-shaped cells closely adjacent to each other are visualized in the apical region; their short dendrites reach the surface and bear cilia (Fig. 3a and g). We did not find any changes in the morphology of 5-HT-ir elements in the late trochophore stage.

In the early D-hinge veliger stage (36 hpf), three 5-HT-ir cells are present within the AO (two flask-shaped and one round, Fig. 3b, b1-b2 and g), and tiny neurites extend from the 5-HT-ir apical cells and run towards the velum. By 52 hpf, long immunopositive neurites extend anterior−dorsally and posterior−ventrally from the AO and run along the ventral sides of the larval body (Fig. 3c and g). Later, two posterior−ventral neurites run along the ventral part of the larval body. The AO consists of three flask-shaped and two round cells, and basal processes of AO neurons are organized into a compact central neuropil (Fig. 3c and c1-c3). Two posterior−ventral neurites from cells of the AO run in parallel along the ventral part of the larval body organizing the ventral nerve cords (VNC) (Fig. 3d and g).

By the late veliger stage (9 dpf), the main 5-HT-ir structures, i.e., the cerebral ganglion and the pedal ganglia, are located along the ventral nerve cords. In addition, small immunoreactive posterior neurons are located near the caudal end of each ventral cord. At this and later stages, CG neurites strongly innervate the velum region (Fig. 3e).

In the later veliger stage (15 dpf), commissures connecting the left and right nerve cords are visible, and as in the previous stage, clearly visible cerebral and pleural parts of fused central ganglia are located on top of the ventral cords (Fig. 3f, f1-2 and g). Peripheral innervation of visceral organs is extensive, including cerebro−pleural ganglia innervation of the velum and pedal ganglia innervation of the foot and the rest of the visceral mass (Fig. 3f and f2).

### Characterization of VAChT antibodies and VAChT-immunoreactivity in *Crassostrea gigas* tissues

**Fig. 4:**
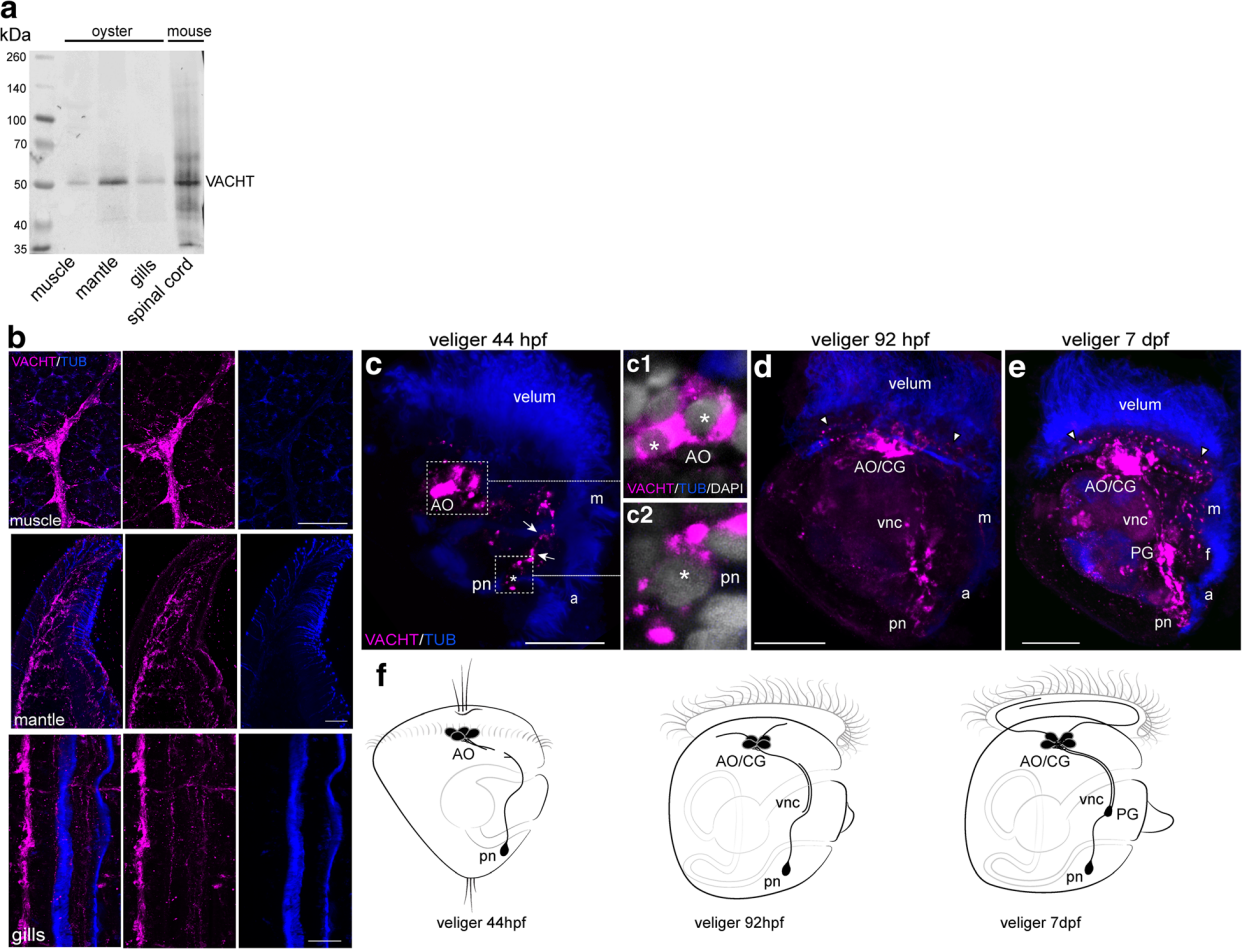
Characterization of VAChT antibodies and VAChT-ir (VAChT, magenta) in oyster, *Crassostrea gigas*, tissues. **a** Western blot of total protein lysates from adult oyster tissue probes stained with goat polyclonal antibodies against rat VAChT. The specific band is detected in all tested oyster tissues as well as in cell lysate from the mouse spinal cord. **b** Double-staining for VAChT/tubulin (VAChT/TUB) of frozen sections of adult oyster tissues. A strong positive signal is detected in the anterior adductor muscle (muscle), mantle, and gills at the structures corresponding to the nerve bundles. **c-e** Confocal images of the larvae stained with VAChT/tubulin, right side view; the anterior is always up. **c** D-hinge veliger. The apical organ (AO) contains two to three cells and their basal fibers. Paired solitary neurons located posteriorly on the right and left sides of the larval body (pn). Each posterior cell sends an anteriorly directed fiber along the ventral edge of the larval body (*arrows*) (an *asterisk* marks the neuron nuclei). **c1** Bodies of two AO neurons; *asterisks* mark the nuclei. **c2** Posterior neuron, an *asterisk* marks the nucleus. **d** In the veliger stage (92 hpf), a strong VAChT-ir signal is detected within the AO/CG complex, and single fibers appear to innervate the velum (*arrowheads*). Immunopositive fibers run in two parallel, ventral cords (vnc), and a solitary posterior cell is visible at the caudal end of each ventral cord. Immunopositive cells appear in the PG. **e** At the 7-dpf veliger stage, VAChT-ir fibers from AO/CG innervate the velum, and fibers from PG innervate the foot (f) and regions around the mouth. **g** Summary diagram of the ontogeny of the VAChT-ir-containing structures in *C. gigas*. Only one lateral side is shown. Additional abbreviations: a – anus, m – mouth. Scale bars =100 μm in **b** and 20 μm in **c**

To demonstrate the specificity of the VAChT and ChAT antibodies, we performed western blot analyses and immunohistochemistry staining of several tissues from the adult oyster. Western blotting indicated the presence of a VAChT-like protein (VAChT-LP) in oyster samples extracted from the muscle, mantle, and gills (Fig. 4a). The VAChT antibody recognized a protein with a molecular weight of approximately 53 kDa, which corresponds to the size of VAChT in the mouse central nervous system [26]. As a positive control for identifying the VAChT band, we used whole cell lysates from mouse spinal cord (Fig. 4a). We also performed double (VAChT/α–acetylated tubulin) immunofluorescence labeling of adult tissues and found VAChT-immunoreactive (VAChT-ir) nerves in all tested adult tissues (Fig. 4b). These results indicate that the commercially available VAChT antibody specifically recognizes the target protein in oyster tissues.

To examine the spatiotemporal distribution of VAChT-ir nerves, we monitored its expression pattern from the trochophore (24 hpf) to veliger (7 dpf) stages. No VAChT-ir elements are observed in trochophores (data not shown); instead, they first appear in cell bodies and processes of the apical cells and in solitary posterior cells at the veliger stage (44 hpf; Fig. 4c, c1-2 and f). Cell processes run toward each other along the ventral edge of the larvae. At the 92 hpf veliger stage, VAChT-ir become more intense within the AO/CG complex, pedal ganglion, and along the caudal portion of the ventral cords, and single fibers appear to innervate the velum (Fig. 4d and f). In the 7-dpf veliger stage, VAChT-ir cells are found in the main larval neuronal structures: the AO/CG complex, ventral cords, PG, and posterior neurons. Immunoreactive fibers appear in the velum, developing foot, and around the mouth (Fig. 4e and f). In contrast, no positive ChAT bands and no immunopositive signal were found in any tested adult or larval oyster tissues (Additional file 2: Figure S2).

### Mutual arrangement of FMRFa-ir, 5-HT-ir, and VAChT-ir

**Fig. 5:**
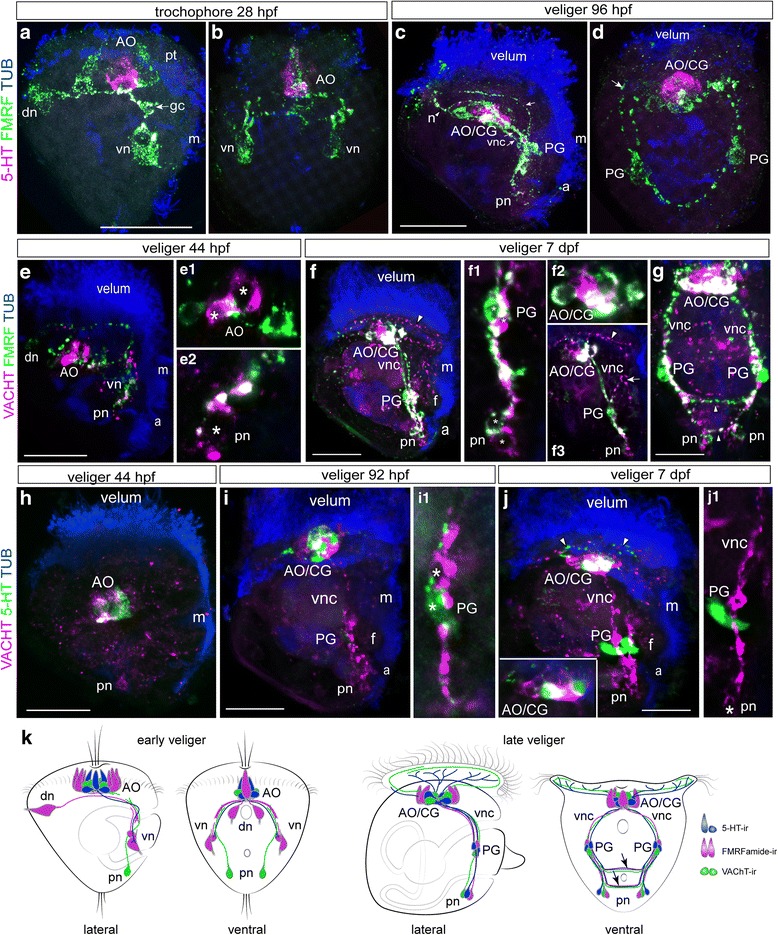
Double staining of 5-HT/FMRFa-ir, VAChT/FMRFa-ir, and VAChT/5-HT-ir nervous elements combined with tubulin immunoreactivity of ciliary structures in *Crassostrea gigas* larvae*.* Neurotransmitter co-localization appears white in all pictures. **a** and **b** Side and front views, respectively, of the alternative expression of FMRFa-ir and 5-HT-ir within early neurons at the 28-hpf trochophore stage. In the apical organ (AO), 5-HT-ir neurons are surrounded by FMRFa-ir neurons. Arrows point to the growth cone of the right dorsal cell (gc). **c** and **d** Alternative expression of FMRFa-ir and 5-HT-ir in cells of the AO/cerebral ganglia complex (AO/CG) at the 96-hpf stage. Only FMRFa-ir cells are present in the pedal ganglia (PG) anlagen. Arrows point to the peripheral process from the PG to the velum, and an *arrowhead* marks the process (n) from the AO/CG to the velum. **e** Alternative expression of VAChT-ir and FMRFa-ir within the early neurons (dn, vn, pn, and AO) in the 44-hpf veliger. **e1** VAChT-ir cells (*asterisks*) are surrounded by FMRFa-ir cells in the AO. **e2** A posterior VAChT-ir cell (pn) sends a process along the FMRFa-ir fiber; an *asterisk* marks the posterior cell nucleus. **f** and **g** Side and front views in the 7-dpf veliger stage demonstrate the presence of both VAChT-ir and FMRFa-ir elements in the AO/CG complex, PG, and posterior neurons (pn). Partial co-localization occurs in the AO/CG neuropil and in the ventral nerve cords. **f1** and **f2** Micrograph demonstrating alternative VAChT-ir and FMRFa-ir expression in PG and AO/CG neurons, their partial co-localization within the AO/CG and the PG neuropil, and the processes of the ventral nerve cords (vnc). **f3** A VAChT-ir process runs from the AO/CG to the velum (*arrowhead*) and from the PG to the foot anlagen (*arrow*). **h** Alternative expression of VAChT-ir and 5-HT in the 44-hpf veliger stage. The AO contains both VAChT-ir and 5-HT-ir neurons, while a posterior neuron (pn) exhibits VAChT-ir only. **i** In the 92-hpf veliger stage, VAChT and 5-HT are alternatively seen within the cell bodies of the AO/CG and are partly co-localized in the neuropil. **i1** Magnification of 5-HT-ir and VAChT-ir neurons in PG. A 5-HT-ir process runs along the VAChT-ir fiber; *asterisks* mark the cell bodies. **j** In the 7-dpf veliger stage, both VAChT-ir and 5-HT-ir fibers emanating from the AO/CG run to the velum (*arrowheads*). **inset:** Alternative expression of VAChT-ir and 5-HT-ir within the AO/CG cell bodies and their partial co-localization within the neuropil. **j1** 5-HT-ir within the PG appears to be adjacent to a VAChT-ir nerve bundle in the ventral nerve cord. An *asterisk* marks the body of the posterior VAChT-ir cell (pn). **k** Summary diagram of the ontogeny of the VAChT-ir, 5-HT-ir, and FMRFamide-ir-containing structures in *C. gigas.* Only one lateral side is shown. Additional abbreviations: a – anus, m – mouth, pt. – prototroph. Scale bars =20 μm

Double-labeling revealed that antibody labeling of the FMRFa-ir and 5-HT-ir neurons does not indicate co-localization within elements of the nervous system at the trochophore stage (Fig. 5a, b). The 5-HT-ir apical neurons are located between the two symmetric FMRFa-ir apical cells, and processes of dorsal FMRFa-ir cells run underneath the AO cells to ventral FMRFa-ir cells that are not 5-HT-positive. In the veliger stage (96 hpf), the 5-HT-ir cells are located centrally within the AO/CG and are surrounded by FMRFa-ir cells. The partial co-localization visible in the neuropil probably appears because of the compact organization of the short cell axons in this region (Fig. 5c, d). At this stage, only FMRFa-ir cells are detected within the pedal ganglia anlagen and along the ventral nerve cords (Fig. 5d and k).

Double-labeling demonstrated the absence of FMRFa-ir and VAChT-ir co-localization within cell bodies in the veliger stages (Fig. 5e and f). In the 44-hpf veliger stage, the VAChT-ir cells are surrounded by FMRFa-ir cells in the AO (Fig. 5e, e1 and k), and small, solitary caudal VAChT-neurons send the process anteriorly along the ventral FMRFa-ir fiber. The locations where FMRFa-ir and VAChT-ir processes intertwine appear partly co-localized (Fig. 5e2 and f1). In the 7-dpf veliger stage, both VAChT-ir and FMRFa-ir are detected in the neurons of the AO/CG complex and PG as well as in the bodies of solitary posterior neurons (Fig. 5f1-3, g, front view and Fig. 5k). Partial co-localization of FMRFamide immunoreactivity and VAChT immunoreactivity within the ventral cords, commissures, and apical neuropil is due to tightly contiguous processes exhibiting different immunoreactivities (Fig. 5f1, f2 and Fig. 5g). Double-labeling indicates the absence of 5-HT-ir and VAChT-ir co-localization within early neurons in the veliger stages. At the 44-hpf veliger stage, the AO contains closely compacted VAChT-ir and 5-HT-ir neurons, while solitary posterior cells only exhibit VAChT-ir (Fig. 5h and k). In the 92-hpf and 7-dpf veliger stages, VAChT and 5-HT are expressed within the cell bodies of the AO/CG, and their basal fibers come into close contact in the neuropil (Fig. 5i, j inset, k). VAChT- and 5-HT are detected within different cell bodies of the PG (Fig. 5i1, j1 and k). In the 7-dpf veliger, VAChT-ir and 5-HT-ir fibers emanating from the AO/CG innervate different portions of the velum (Fig. 5k). Only VAChT-ir, but not 5-HT-ir, is detected in the caudal posterior cells at the 7-dpf stage (Fig. 5j1).

### Neuromorphology of the pediveliger and peripheral innervation of larval tissues

**Fig. 6:**
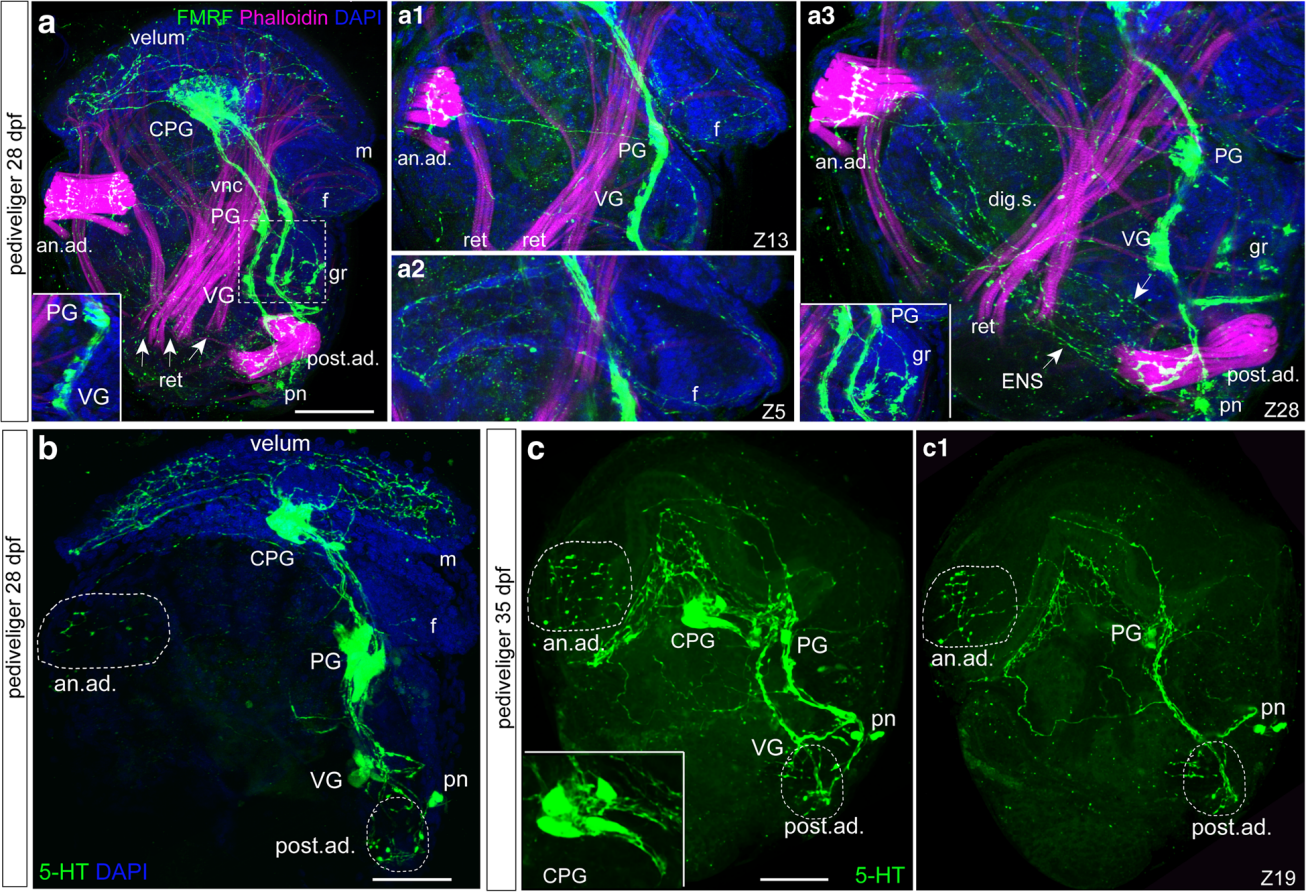
FMRFamide-immunoreactivity (FMRFa-ir) combined with phalloidin staining and serotonin-immunoreactivity (5-HT-ir) in the pediveliger stage (28 dpf) of *Crassostrea gigas*. Magenta—muscles, phalloidin; blue—nuclei, DAPI. The apical pole is always upward, and the ventral side is on the right. **a** General view of FMRFa-ir in the 28-dpf pediveliger stage with the cerebro-pleural ganglion (CPG), paired ventral cords (vnc), pedal (PG), and ventral (VG) ganglia located along the vnc. Note the absence of special cerebro-pedal cords separated from the vnc bundles. Solitary posterior neurons (pn) are visible at the most caudal part of each vnc. Peripheral innervations of the velum, gill rudiment (gr), anterior and posterior adductor muscles (an.ad. and post.ad.) and body wall are visible. **Inset:** High magnification of the PG/VG region exhibiting a chain of FMRFa-ir cells within the VG rudiment. **a1** Innervation of anterior adductor muscle (an.ad.) with fibers emanating from the PG. Retractor muscles (ret) are innervated from the VG. **a2** A network of thin FMRFa-ir fibers in the foot (f). **a3** The FMRFa-ir network along the digestive system (*arrowheads*, dig.s.) forms a network of the enteric nerve system (ENS). The posterior adductor (post.ad) is innervated by a thick fiber from the VG. **inset:** High magnification view of the developing gill rudiment (gr) with two parallel, branched fibers emanating from the PG. **b** General view of 5-HT-ir in the 28-dpf pediveliger stage with CPG, pedal (PG), and ventral (VG) ganglia located along the ventral nerve cords. Solitary posterior neurons (pn) are visible near the most caudal part of each vnc. Peripheral innervations of the velum and anterior and posterior adductor muscles (an.ad. and post.ad.) are visible. **c** General view of 5-HT in the 35-dpf pediveliger with a well-developed cerebro-pleural ganglion (CPG), and pedal (PG) and ventral (VG) ganglia. **Inset:** High magnification of the CPG. **c1** The anterior adductor and velum are innervated from the CPG and PG. The posterior adductor is innervated from the ventral ganglia. Scale bars =50 μm

In the pediveliger stage (28 dpf), FMRFamide-ir cells are found in the cerebro-pleural ganglia, pedal ganglia, newly formed visceral ganglia, and solitary posterior neurons situated at the caudal end of the paired ventral cord (Fig. 6a). Processes emerging from the cerebro-pleural ganglia strongly innervate the velum. In addition, combining FMRFamide-immunostaining with phalloidin clearly reveals innervation of the muscles; thick nerves from the pedal ganglia innervate the anterior adductor muscle and rudimentary gill (Fig. 6a1, a3 and a3 inset). The retractor muscles and the foot are innervated by the pedal ganglia (Fig. 6a1-2). A network of FMRFa-ir fibers is present in the digestive system, forming the enteric nerve system (Fig. 6a2 and a3). Thick nerves from the visceral ganglia innervate the posterior adductor and body wall (Fig. 6a3).

5-HT immunostaining can be observed in the main ganglia (cerebro-pleural, pedal, and visceral). FMRFa-ir and 5-HT-ir cells are equally present within the cerebro-pleural ganglia, while 5-HT-ir cells are prevalent within the pedal ganglia (Fig. 6b). In the 35-dpf pediveliger, the cerebral and pleural regions of the cerebro-pleural ganglion are clearly visible (Fig. 6c inset), and the velum receives rich innervations from the cerebro-pleural and pedal ganglia (Fig. 6b, c and c1). Nerves emerging from the pedal and visceral ganglia show intense varicose ramifications within the anterior and posterior adductors, respectively (Fig. 6c and c1).

## Discussion

### Comparative neuromorphology of Bivalvia larvae

**Fig. 7:**
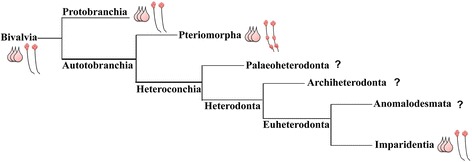
Phylogenic tree of major bivalve lineages based on González et al. [1], revealing ground patterns based on morphological data of neurogenesis of bivalves that have been investigated. Pink cells are 5-HT-ir cells of the AO in the studied species. Ventral nerve cord (VNC, black parallel lines) with red ganglia (top is the cerebral, middle is the pedal, and bottom is the ventral ganglion). Protobranchia (non-published data), Pteriomorpha, and Imparidentia show three flask-shaped cells in the AO. Within Pteriomorpha mussels and oysters possess a VNC with three paired ganglia, while Protobranchia and Imparidentia have a VNC with only a cerebral ganglion

Recent phylogenomic RNA-seq data approaches have provided sound information on the phylogenetic relationships between major bivalve lineages. Based on these phylogenomic data, it is believed that Bivalvia are arranged into five clades (Protobranchia, Pteriomorpha, Palaeoheterodonta, Archiheterodonta, Euheterodonta) [1] (Fig. 7).

Data on bivalve neurogenesis are limited and often restricted to accounts of single species and stages of development performed by light microscopy [8, 27–29]. Comparative analysis of immunocytochemical data on bivalves are still scare and restricted to the analysis of the development of serotonin-, FMRFamide- and catecholamine-ir, small cardioactive peptide-like nerve elements in larvae of the mussels *Mytilus trossulus* [5] and *Mytilus edulis* [4], scallop *Placopecten magellanicus* [4], oyster *Crassostrea virginica* [6], clam *Spisula solidissima* [15] and mussel *Dreissena polymorpha* [16]. Unfortunately, no published neurogenesis data have been reported for the Protobranchia, Palaeoheterodonta, Archiheterodonta, and Anomalodesmata clades. This lack of data makes it difficult to identify the ground pattern of the larval nervous system and to clarify phylogenetic relationships between bivalve species. Nevertheless, using published, non-published data and novel neuroanatomical studies presented here, we have inferred a ground pattern of the larval nerve system inherent to some Bivalvia clades: Pteriomorpha and Euheterodonta (Fig. 7).

### Cell composition of apical organ in bivalves

The apical organ (AO) is the most conserved larval sensory structure and has plesiomorphic features of high importance for studies of animal evolution and relationships. In the bivalve species (Pteriomorpha and Imparidentia), the AO develops from early larval stages (early trochophore) and consists of ciliated flask-shaped and rounded 5-HT-ir as well as flask-shaped FMRFamide-ir cells [5, 16].

**Fig. 8:**
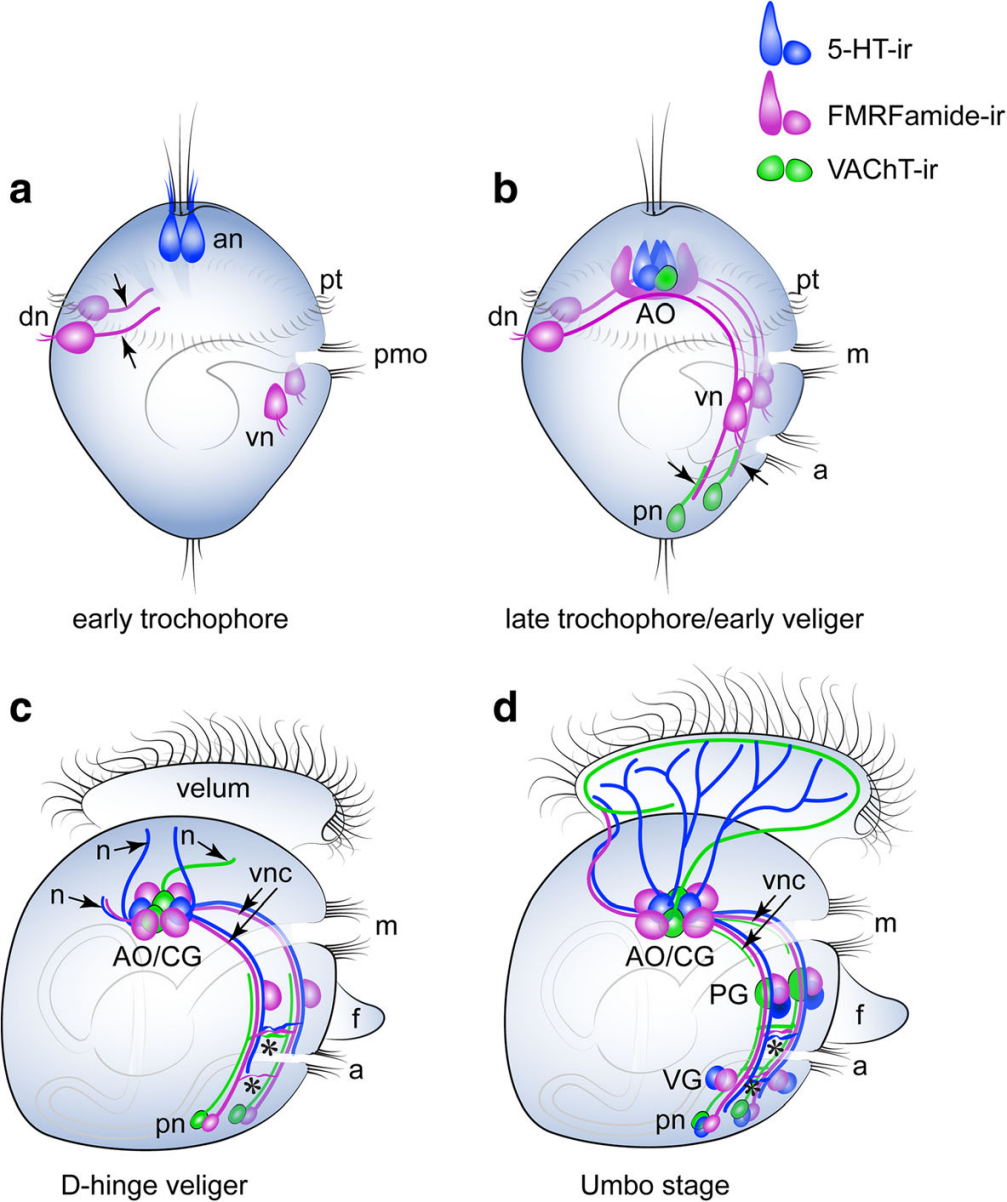
Summary schematic of the early neurogenetic events of the Pacific oyster, *Crassostrea gigas.* Serotonin-immunoreactive (5-HT-ir) elements are blue; FMRFa-ir elements are violet; and VAChT-immunoreactive (VAChT-ir) elements are green. The apical pole is always upward, and the ventral side is on the right. **a** At the early trochophore stage, only 5-HT-ir cells are present in the apical region of the episphere. Apical neurons (an) have a flask shape and bear cilia. Paired dorsal (dn) and ventral neurons (vn) located in the hyposphere region express FMRFa-ir. The dorsal cells have a ciliated sensory dendrite and an axon extending towards the ventral cells (*arrowheads*); VAChT-ir elements are absent. **b** By the late trochophore/early veliger stage, the axons of the dorsal cells pass a group of ventral cells to reach the caudal region of the larvae. VAChT-ir neurons appear posteriorly (pn) and send axons anteriorly along the FMRFa-ir processes of the dorsal cells. Thus, the paired ventral nerve cords are established by early FMRFa-ir and VAChT-ir nerve elements. FMRFa-ir and VAChT-ir flask-shaped cells are incorporated into the apical organ (AO), and AO neurons are located between the ventral cords but have no connections with them. **c** In the D-hinge veliger stage, solitary FMRFa-ir neurons (pn) appear posteriorly near the posterior VAChT-ir cells. Axons of apical, ventral, and posterior neurons grow along the ventral cords. Two commissures (*asterisks*) connect the left and right ventral cords (vnc). The AO is located between the two cerebral ganglia forming the AO/CG-complex. 5-HT-ir, VAChT-ir, and FMRFa-ir neurites (*arrowheads*, n) extend from the AO/CG neuropil. **d** In the umbo stage, the pedal (PG) and visceral ganglia (VG) adjoin the longitudinal ventral cords. No specific cerebro-pedal cords can be separated within the bands of the ventral cords. The CPG, PG, and posterior group contain FMRFa-ir, 5-HT-ir, and VAChT-ir neurons, while the VG only contains FMRFa-ir and 5-HT-ir cells. Both ventral cords and commissures contain FMRFa-ir, 5-HT-ir, and VAChT-ir neurites. 5-HT-ir and VAChT-ir processes extending from the CG richly innervate the ciliated velum. 5-HT-ir and FMRFa-ir neurites innervate the velum anterior edge. Abbreviations: a – anus, an – apical neuron, CG – cerebral ganglia, dn – dorsal neurons, m – mouth, n – peripheral neurites, PG – pedal ganglia, pmo – presumptive mouth opening, pt. – prototroch, VG – visceral ganglia, vn – ventral neurons, vnc – ventral nerve cords

**Fig. 9:**
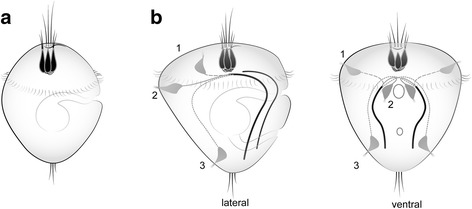
Schematic presentation of the ground structures of the nervous system in Bivalvia and Mollusca based on morphological data of neurogenesis in studied species. **a** suggested ground pattern of AO of the last common ancestor of Bivalvia consist of three flask-shaped cells. **b** the most conserved nervous structures in Mollusca are an AO consisting of three flask-shaped cells and a paired ventral nerve cord (black lines). Note, the neurites of pioneer peripheral sensory cells are located in the episphere (1st scenario for Mytillus trossulus, Bivalvia) or hyposphere (located dorsally (2nd scenario for oyster) or caudally (3rd scenario for chitons and gastropods); all trace the future VNC. Thus, the position of first sensory cells in larvae does not matter, but the route that the neurites make is necessary for positioning of the VNC later

The AO of *Crassostrea gigas* trochophores contains two and later three 5-HT-ir flask-shaped cells and two rounded cells in oyster veligers (five cells in total; Fig. 8). Interestingly, the AO of *Mytilus trossulus* larvae consists of three 5-HT-ir flask-shaped cells in trochophores and five 5-HT-ir cells in veligers which later become a part of the emerging cerebral ganglion [5]. The same number of flask-shaped 5-HT-ir cells was also detected in the clam *Spisula solidissima* [15]. Findings on the number of 5-HT-ir flask-shaped cells of AO in *Dreissena polymorpha* are however contradictory: one study demonstrated that the AO consists of three 5-HT-ir flask-shaped cells (Voronezhskaya, personal data), but another described four 5-HT-ir flask-shaped cells in the AO [16]. *Nucula tumidula* (Protobranchia) were reported to have three 5-HT-ir flask-shaped cells in pericalymma larvae [30]. Briefly, we can conclude that three 5-HT-ir flask-shaped cells, as are present in the AO in larvae of the Protobranchia, Heterodonta, and Pteriomorpha species, are more common and are possibly a ground attribute in Bivalve larvae studied (Figs. 7 and 9).

In addition to serotonin-ir cells, the AO of *Crassostrea gigas* larvae contain two, four, and later six FMRFamide-ir flask-shaped cells in veliger larvae (Fig. 8). Five FMRFamide-ir cells were detected in *Mytilus trossulus* AO at D-veliger stage [5]. It is possible that this large number of FMRF-ir cells in the AO represent a common morphological criterion for bivalves or Pteriomorpha species. However, without additional information of the FMRFamide containing cells within AO in Palaeoheterodonta, Archiheterodonta, and Anomalodesmata clades, it is difficult to reconstruct the ground pattern of the nervous system for Bivalvia.

### Ventral nerve cord development and main ganglia

A distinctive feature of early neurogenesis of the oyster *C. gigas* is that the first-appearing peripheral sensory FMRF-ir cells are located posttrochally at the dorsal and ventral sites of the larvae (Fig. 8). All cells have short sensory dendrites and the long axons form a scaffold of two paralleled ventral nerve cords (VNC), which will be followed by growing axons of caudally positioned VAChT-expressing cells. Such morphology is very prominent and is characteristic for most of the pioneer neurons described in the development of other trochophore animals (Nezlin and Voronezhskaya [31]). As in other described trochophore larvae, both early dorsal and ventral groups are probably transient and resorbed after fulfilling their routing function. In a previous investigation of *Mytilus trossulus* larvae, all early peripheral FMRF-ir cells were found in the episphere at the trochophore stage, with the FMRFa-ir pioneering axons projecting to the ventral part of the larvae to form the VNC in the veliger [5]. Thus, in both oyster and mussel larvae, FMRFa-ir processes of early cells track in a dorso-ventral direction to form the anlagen of the VNC, which will be followed by neurites of cells differentiating later within the AO and main ganglia rudiments. Later commissures connect left and right longitudinal cords producing a rope-ladder-like (segmented) ventral nerve structure. Therefore, while there appear to be differences in the number and position of early peripheral FMRFa-ir cells and 5-HT-ir cells between bivalves and in spite of species-specific early patterns of neurodevelopment, the same final result of an AO and paired VNC is realized (Fig. 9).

Interestingly, serotonin Abs also mark the VNC in oyster with two commissures appearing at the late veliger stage, whereas serotonin immunostaining is restricted to the cerebral ganglion in mussel *M. trossulus* veliger/pediveliger [5]. In contrast to *M. trossulus*, *Dreissena polymorpha* has a 5-HT-ir VNC and cerebro-visceral connectives starting from the veliger stage [16]. In addition, in the oyster *Crassostrea virginica,* small cardioactive peptide (SCP-like neuropeptide) also label the VNC with two commissures at the late veliger stage [6]. These also confirm the VNC as a common neuronal morphological structure for all investigated Bivalvia larvae (Fig. 9) and a feature for comparative analysis within lophotrochozoan clades.

All larval ganglia appear along the VNC as paired or fused (epiathroid) clusters in late veliger and pediveliger larvae. The maturation of the anlagen of the cerebral ganglion (CG) is characterized by loss of flask morphology of FMRFa-ir and 5-HT-ir cells in the AO and the appearance of abundant serotonin innervation of the velum in the late oyster veliger (Fig. 8). Whether the apical organ cells are precursors of neurons of the CG remains unclear. Based on our data, we cautiously assume that cells of the AO are incorporated within the CG and became a part of the juvenile oysters CG. This suggestion is supported by data from *M. trossulus* neurogenesis representing continuity of cilia flaked-shaped cells of the AO into the cerebral ganglion [5], and by recent reconstructions of the serotoninergic nervous system in *Dreissena polymorpha* [16].

The anlagen of the pedal ganglia (PG) appears along each ventral cord in the region of the developing foot in the oyster *Crassostrea gigas* and mussel *M. trossulus* veliger [present data and 5]. It seems that the precursors of PG neurons are rounded FMRFa-ir and 5-HT-ir ventral neurons appearing in early and late oyster veligers (Fig. 8). PG in the late veliger of *Crassostrea virginica* contains SCP-like neuropeptide-positive cells [17]. In *M. trossulus,* neurons of PG include catecholamine-ir and FMRFa-ir cells, whereas in *D. polymorpha* and *Spisula solidissima,* PG neurons were not detected at any developmental stages using 5-HT and tubulin antibodies. If true, the absence of pedal neurons could be an apomorphic character of the Heterodonta clade. However, the absence of PG neurons (or positive immunostainings) in *Spusula solidissima* and *D. polymorpha* veligers is unconvincing, because both species have a foot that exhibits activity in pediveliger larvae [32, 33]. Future studies of neurogenesis using FMRFamide and VAChT Abs, as introduced herein, as well as other neuronal markers are needed to determine conclusively if there is a PG neurons in these species.

Visceral ganglia (VG) are the last ganglia appearing in oyster development (Fig. 6). These paired ganglia contain FMRFamide-ir and 5-HT-ir neurons. In *M. trossulus,* visceral neurons express FMRFamide-ir and catecholamine-ir, but not 5-HT-ir. VG in *D. polymorpha* was not found in the veligers (144 hpf), because of absent or belated ganglia at this stage [16]. The data on the presence or absence of the VG in *S. solidissima* at the early veliger stage (48 hpf) is needed to verify the neuronal ground pattern for Bivalvia. Thus, VG is a common morphological structure appearing at late developmental stages and is one of the main criteria defining the Pteriomorpha species.

### VAChT-ir nerve elements

Using a VAChT antibody, this study provides the first characterization of the acetylcholine (ACh)-ir nervous system in bivalves. We found VAChT-ir in the AO, posterior neurons and, later, in the cerebral, pedal ganglia, and VNC (Fig. 8). Acetylcholine has been detected in the larvae of *M. edulis* using a histochemical technique, as well as in the larvae of the annelid *Platynereis dumerilii* by whole-mount in situ hybridization (WMISH) [3, 10, 34–36]. Acetylcholinesterase-positive ciliary sensory-like cells have been identified as the earliest rudiments of the pedal ganglia and primary byssus glands of mussels by Rainery and Ospovat. In addition, Raineri identified three pairs of posttrochal ganglia rudiments, which were considered to be the visceral, parietal, and pleural ganglia located along the longitudinal nerve cords in the trochophore larva. The author claimed that the cerebral ganglia differentiate adjacent to AO, and the nerve network appears at the velum after the differentiation of the first pedal nerve rudiments. While histochemical method used in this study did not allow unequivocally identify nerve cells and their processes, the main pattern of the mussel nervous system was similar to that observed later using immunochemical markers. It is known that the early larval neurons in the annelid *P. dumerilii* express the cholinergic marker VAChT, as determined by WMISH at the trochophore stage, and VAChT expression later expands to the VNC and brain, as well as the neurons of the peripheral nervous system (which is associated with the appendages) [35, 36]. Further research on Bivalvia neurogenesis, with particular focus on VAChT-ir structures, is required in order to understand the cell composition of the AO and central and peripheral nervous systems in veligers, and to expand understanding to their role in the regulation of physiological functions.

### Innervation

Innervation of the velum by 5-HT-ir and FMRFamide-ir neurites (which form the velar nerve ring or plexus pattern) originating from the AO/CG has been demonstrated in various gastropods [37–40] and bivalves [2, 5, 41]. Early veligers of *Crassostrea gigas* present dorsal velum innervation by both FMRFamide-ir and 5-HT-ir neurites projecting from the AO/CG. VAChT-ir neurites form the neural ring from the ventral side of the AO. Recent data show that *Dreissena polymorpha* has 5-HT-ir dorsal velum innervation [16] very similar to what is observed in *C.gigas*. Later, *C. gigas* larvae demonstrate an abundant 5-HT-ir plexus-like innervation pattern similar to that detected in the pediveliger of *Mytilus edulis* [4]. Catecholaminergic cells and their neurites are also found in the velum of the veligers and pediveligers of *Placopecten magellanicus*, *M. trossulus*, and *M. edulis* [4, 5]*.* In addition, SCP-like peptides label processes in velum that originate from cerebral ganglion (CG) [6]. Thus, the velum of Bivalvia larvae is under neuronal control by different neuropeptides and neurotransmitters in veligers and receives abundant innervation in pediveligers of Pteriomorpha species. Unfortunately, there are not enough data for comparative morphological analysis of velum innervation patterns among other Bivalve clades and future investigations are needed, including investigations of Hererodonta species (*D. polymorpha* and *S. solidissima,)* in late developmental stages.

We found that smooth adductor muscles are innervated by serotonergic and FMRFamidergic neurons in the pediveliger larvae of the oyster *C. gigas.* The same innervation patterns were shown in *Mytilus trossulus* pediveligers [23]. In *C. gigas* and *M. trossulus,* FMRFamide-ir fibers originate from the PG and innervate the anterior adductor in pediveligers, while processes from the visceral ganglia innervate the posterior adductor in *C. gigas*. 5-HT, as a key regulator catch-contraction of adult adductors, was detected in oyster pediveligers innervating anterior and posterior adductors. Interestingly, 5-HT innervation was not detected in the anterior adductor of the mussel *M. trossulus* pediveliger [5, 23]. Further research on neuromuscular interactions in pediveligers is required for an understanding of the neuronal control of catch contractions and innervation patterns after metamorphosis.

Here, we made the novel observation of FMRFamide immunoreactivity throughout the enteric nervous system, including the digestive system, and innervations of paired gill rudiments in oyster pediveligers. We suggest the FMRFamide-ir cell bodies and fibers either originate from the VG or arise independently of ganglia (autonomous or local genesis) from ganglia manner. It should be noted, that 5-HT does not innervate these organs in late larvae. However, VAChT-ir neurites also participate in innervation of the mouth region in veligers.

To summarize the common characteristics of the bivalves studied herein: (1) bivalve larvae show an apical organ composed of three 5-HT-ir flask-shaped cells, which is an ancestral feature of three to five subclasses of Bivalvia, and two 5-HT-ir round cells that are detected in Pteriomorpha and Imparidentia; (2) the AO in Pteriomorpha larvae show six FMRFamide-ir flask-shaped cells (apomorphic feature); (3) in the post AO-period, the larval nervous system of bivalves have ganglionic-like structure characteristic of the adult nervous system; (4) a paired ventral nerve cord is a common feature (plesiomorphic) of the bivalve larvae investigated herein (Figs. 7 and 8). We suggested these features were inherent to the larva of the last common bivalve ancestor (LCBA). To understand additional morphological criteria, such as the VAChT-ir nervous system, innervation patterns, enteric nerve system, more data are necessary for all clades of Bivalvia.

### Comparison of larval neuromorphological structures in Mollusca

Phylogenomic analysis has divided all Mollusca into two main Subphyla: Aculifera, which comprises Neomeniomorpha, Chaetodermomorpha, Polyplacophora, and Conchifera, which comprises Monoplacophora, Cephalopoda, Scaphopoda, Gastropoda, and Bivalvia [42]. Despite extensive molecular and morphological investigations of conciferan taxons, our understanding of their positions on the phylogenetic tree and their interrelationships remain unclear. Here, we detail the main morphological criteria.

#### Apical organ (AO) in Mollusca

Among larval structures, the AO is a temporary sensory organ found in the early stages of development among phylogenetically diverse animal groups that presumably acts as a chemosensory structure that resorbs before or after metamorphosis. An AO consisting of flask-shaped cells has also been reported in most other lophotrochozoans, such as annelids, nemerteans, ectoprocts and the brachiopods, and phoronids [43–49]. 5-HT- and/or FMRFamide-ir cells can be found in the AO of both Aculifera and Conchifera groups of Mollusca, but their morphology (round, flask-shaped cells, cilia) and their number are variable among mollusc groups. We found/hypothesize that the presiomorphic feature in bivalves is three 5-HT-ir flask-shaped cells in the AO, which has also been described for basal (Protobranchia) and more advanced groups (Euheterodonta). The same number of 5-HT-ir flask-shaped cells in the AO was found in basal gastropod molluscs, patellogastropods *Tectura scutum* [50], and *Lottia* cf. *kogamogai* [51]. Moreover, in addition to flask-shaped cells in the AO, *Lottia* cf. *kogamogai* have two 5-HT-ir round cells, as is observed in the *Crassostrea gigas* AO. The same cellular composition of the AO is present in the larval abalone *Haliotis kamtschatkana,* which is a Vetigastropoda [52]. In opistobranch gastropod larvae, such as *Aplysia californica* (Anaspidea) [37, 53], *Aeolidiella stephania* and *Phestilla sibogae, Berghia verrucicornis, Melibe leonina,* and *Tritonia diomedea* (Nudibranchia) [39, 54, 55], three 5-HT-ir flask-shaped and two non-sensory round cells were detected. The same cellular composition of the AO (three flask-shaped + two round 5-HT-ir cells) was found in the caenogastropod *Euspira lewisii* (Naticoidea) [40] while other species of this clade have differences in the AO cell composition (3 + 3; 2 + 2; 1 + 2), [55]. Although data on FMRFamide-ir cells of the AO are scant for gastropods, bivalves and other clades of molluscs, the number of FMRFamide-ir cells in the AO corresponds to those in some caenogastropods and nudibranchs [38, 55]. Therefore, most gastropod groups have three 5-HT-ir cells in the AO with or without round cells and show a similar arrangement and number as that in Bivalvia AO found in larvae of the protobranchs, pteriomorphs, and imparidents (Fig. 9).

Larval neuromorphology of bivalves and scaphopods (*Antalis entails*) have fewer similarities in the 5-HT-ir system: the number of 5-HT-ir flask-shaped cells in *Antalis entails* larva start from two and reach four cells in the AO (plus two lateral cells) [56]. This number of serotonin cells was found in Bivalvia and gastropods larvae only in early trochophores. There are no published data on the AO cell composition in Monoplacophora. Thus, the ground pattern of AO inherent to Conchifera species’ sensory organ includes two to three flask-shaped 5-HT-ir cells.

Among Aculifera taxons, the most studies Polyplacophora species are *Mopalia muscosa* and *Ischnochiton hakodadensis* [18, 57], which have 8–10 flask-shaped 5-HT-ir cells in the AO. These cells are surrounded by dorsal and ventral cells, and six FMRFamide-ir AO cells and peripheral cells [57]. However, early stages of *Mopalia muscosa* (55 hpf) clearly show three flask-shaped 5-HT-ir cells in the AO and additional peripheral sensory cells [18, 58]. In the Neomeniomorpha (Solenogastres) *Wirenia argentea* and *Gymnomenia pellucida,* the AO contains only two 5-HT-ir cells and two FMRFa-ir cells [59]. These data support there being a simple AO structure in the aculiferan ancestor, and are contrary to postulates of a complex structure of the AO as an ancestral condition for Aculifera (8–10 cells in polyplacophores). For the most complete comparative analysis of neurogenesis in bivalves plus Aculifera and Conchifera and to reconstruct the putative ground pattern of the last common ancestor of Mollusca (LCAM), further detailed and high-quality studies of Neomeniomorpha and Chaetodermomorpha are required.

### Morphology of larval nervous system and reconstruction of possible ground pattern

Comparative morphology and recently emerging genomic data continue to be important tools in our efforts to determine the putative pattern of the Last Common Ancestor of Bivalves (LCAB), Conchifera (LCAC), Aculifera (LCAA), and Last Common Ancestor of Mollusca (LCAM), and to better define phylogenetic relationships between molluscan clades. Comparative analysis of larval neuronal structures showed that Bivalvia larvae have striking similarities in organization and arrangement of the AO (three flask-shaped cells), the position of the main ganglia (cerebral/pleural, pedal, and visceral) along the VNC, and the presence of commissures forming a rope-ladder-like (segmented) nervous system. These features are shared by gastropod larvae with minor modifications (additional ganglia) and are a unifying features of the node-base group Pleistomollusca. Thus, morphology-based analysis supports phylogenomic data claiming that Bivalvia and Gastropods are sister-taxons [60] having a common ancestor (Pleistomollusca ancestor).

The position of Scaphopoda on the phylogenic tree of Conchifera is not clear. Scaphopoda are considered a potential sister group of Pleistomollusca [60] and Bivalvia (Diasomal concept [17, 61]. There may also be a cephalopod-scaphopod relationship [42]. The larval nervous system of scaphopods is ganglion type with a ventral (pedal) nerve cord [56]. From the above, the nervous system of the LCAC presumably had a paired ventral nerve cord with ganglia located along it. The morphology of the LCAA remains elusive and developmental evidence of neurogenesis from recent aplacophoran representatives is still lacking. Popyplacophora and Neomeniomorpha species show differences in cellular composition of the AO and ganglia, as well as in order of appearance of the nerve cords [57–59]. However, despite these differences, we can conclude that the ground pattern of the nervous system of the LCAA is an AO containing two to three 5-HT-ir flask-shaped cells (2 flask-shaped cells in Neomeniomorpha, as we do not take into account alpha-tubulin ciliary staining [59]), three flask-shaped cells at early stages of development [58], and a paired VNC with commissures (segmented) and perikarya positioned along it. If we consider that the polyclocophoran AO of Aculifera and Mollusca generally consists of 8–10 5-HT-ir cells as a basal pattern, then secondary simplification of the cellular composition of the AO in Neomeniomorpha and all Conchifera clades is a general evolutionary trend for Mollusca. To determine the LCAA with greater accuracy and to finally shed light on molluscan ancestor neuromorphology, future investigations on mollusc neurogenesis are needed.

The question of the origin of the VNC as a ground trait for the LCAM is one the main and most interesting question in the evolution of bilaterians. In the oyster *C. gigas,* we found that FMRFamide-ir sensory peripheral cells appear posttrochaly and send their neurites from the dorsal to ventral side of early larvae, thereby playing the role of a scaffold for the VNC (Fig. 9). Pioneer peripheral cells and their neurites in the primordium of the VNC were detected in several lophotrochozoa, with the cell bodies located in the episphere in bivalves (scenario 1 on Fig. 9 [5]), in the hyposphere (dorsally) in annelids and bivalves (scenario 2 on Fig. 9 ([31, 62–64], present data)), and in the hyposphere (caudally or caudo-laterally) in gastropods and polyplacophores (scenario 3 on Fig. 9 [57, 65, 66]). Important, the primary location of the sensory pioneer neurons in larvae is largely irrelevant, while the way of their neurites will ultimately mark a position of VNC, albeit through different pathways (dorso-ventral direction or posterior-anterior direction). Moreover, the presence of a similar dorsoventral arrangement of transcription factors along the nerve cords of Spiralia supports the hypothesis of ancestrality of the VNC in Bilateria [67].

## Conclusions

Here, we described the dynamics of neurogenesis during the development of the Pacific oyster *Crassostrea gigas* using 5-HT and FMRFamide antibodies, as well as VAChT antibodies for detection of cholinergic neurons (a first in bivalves). We conducted detailed neuroanatomical and axon tracing studies through the early trochophore and veliger stages until the pediveliger stage. Comparative morphological analysis of *Crassostrea gigas* neurogenesis with other Bivalvia, revealed common conserved characteristics as well distinctions in early neuronal specialization of the FMRFamide-ir and 5-HT-ir cells in trochophore and veliger larvae. We conclude that the sensory AO in bivalves, as well as in Conchifera, consists of three flask-shaped cells, and that the larval nervous systems are of the ganglion type and have paired VNC with commissures. We propose that axons of early peripheral neurons laying down the pathway for VNC, which is the most conservative larval structure. Our morphological data support phylogenomic data indicating a closer Bivalvia-Gastropoda sister group relationship than the Bivalvia-Scaphopoda (Diasoma) group relationship and raise questions about the validity of the Tetraneuralia concept for bivalves.

## Additional files

Additional file 1: **Figure S1.** Ciliation in *Crassostrea gigas* visualized with anti-acetylated tubulin. a and b: External ciliation. c-e: Optical sections through the middle of the larval body. a: Ciliated blastula stage. b: The trochophore stage with a prominent prototroch (pt) ring. A presumptive mouth opening (pmo) is located on the ventral side underneath the prototroch. c: The late trochophore stage possesses a well-developed prototroch and telotroch, and the digestive system consists of a ciliated mouth (m), esophagus (es), and a digestive mass (DM) as an anlagen of the stomach. c: The early veliger stage is the first feeding stage with a well-developed digestive system including a ciliated mouth (m), esophagus (es), differentiated stomach (st), intestine (in), and anus (a). The differentiated foot (f) is located between the mouth and anus. e: Ciliation in the late veliger is similar to that in the previous larval stage. Scale bar = 20 μm. (TIFF 2302 kb)

Additional file 2: **Figure S2.** Specificity of ChAT antibodies in adult tissues of *Crassostrea gigas* and expression of ChAT-ir in nervous elements. a: Western blot of total protein lysates from adult oyster tissue probes stained with goat polyclonal antibodies against rat ChAT. No specific band is detected in the adult oyster tissues. The positive signal only corresponds to a protein band with a molecular weight of 69 kDa in the cell lysate of mouse spinal cord. b: Immunostaining of frozen sections of adult oyster tissues with ChAT/TUBULIN antibodies show the absence of a positive ChAT-ir signal in all tested tissues. c: Confocal image of the 7-dpf veliger stained with ChAT/TUBULIN antibodies shows no positive inner structures. The signal observed along the shell edge and in the center of the larval body is likely non-specific fluorescence. Scale bar = 100 μm in b and 50 μm in c. (TIFF 2992 kb)
