# Supplementary figures and images for: Nervous system development in the Pacific oyster, Crassostrea gigas (Mollusca: Bivalvia)
Source: Front Zool. 2018 Apr 11;15:10. doi: 10.1186/s12983-018-0259-8 (PMC5896133; doi:10.1186/s12983-018-0259-8)

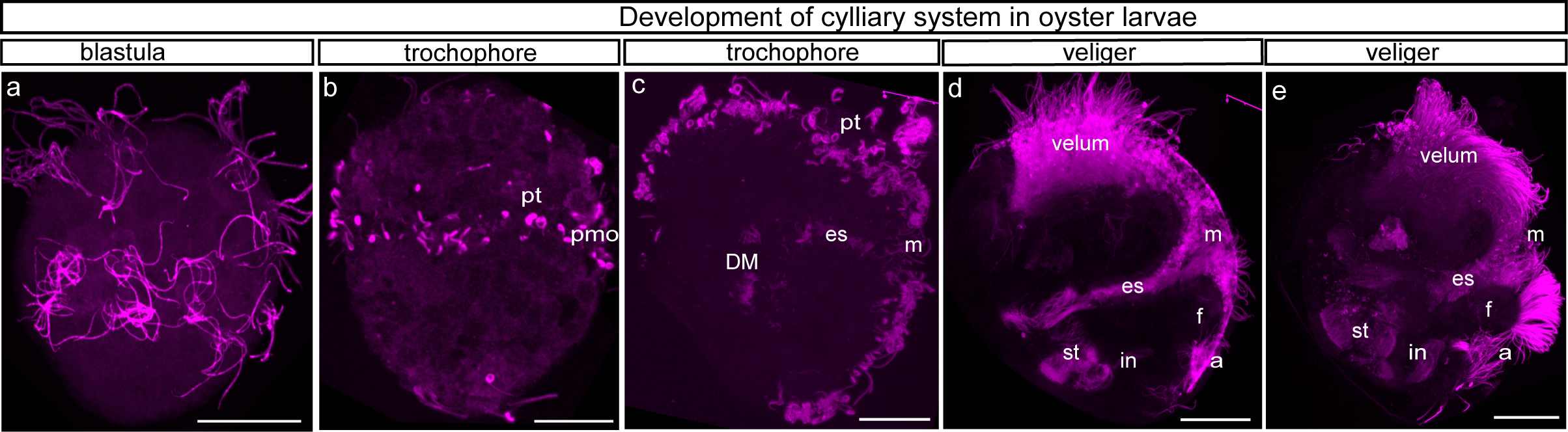

Supplement: Supplementary file 1 — Figure S1. Ciliation in Crassostrea gigas visualized with anti-acetylated tubulin. a and b: External ciliation. c-e: Optical sections through the middle of the larval body. a: Ciliated blastula stage. b: The trochophore stage with a prominent prototroch (pt) ring. A presumptive mouth opening (pmo) is located on the ventral side underneath the prototroch. c: The late trochophore stage possesses a well-developed prototroch and telotroch, and the digestive system consists of a ciliated mouth (m), esophagus (es), and a digestive mass (DM) as an anlagen of the stomach. c: The early veliger stage is the first feeding stage with a well-developed digestive system including a ciliated mouth (m), esophagus (es), differentiated stomach (st), intestine (in), and anus (a). The differentiated foot (f) is located between the mouth and anus. e: Ciliation in the late veliger is similar to that in the previous larval stage. Scale bar = 20 μm. (TIFF 2302 kb) [file 12983_2018_259_MOESM1_ESM.tif]

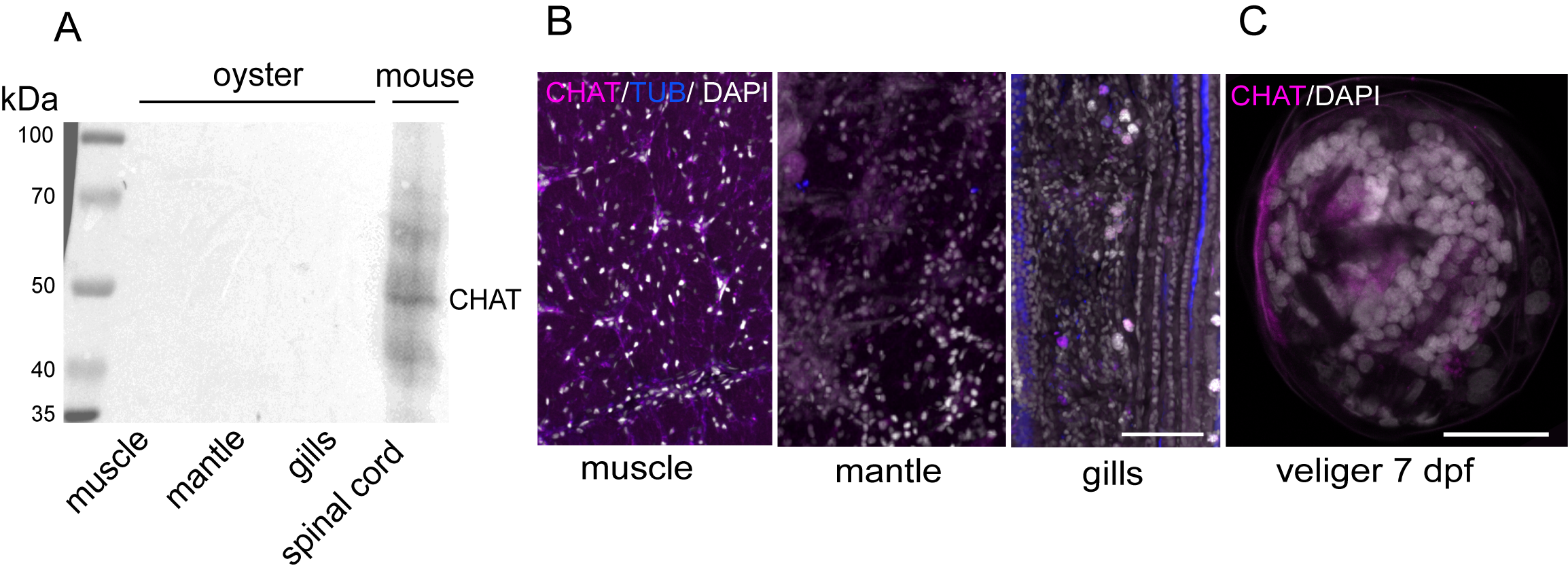

Supplement: Supplementary file 2 — Figure S2. Specificity of ChAT antibodies in adult tissues of Crassostrea gigas and expression of ChAT-ir in nervous elements. a: Western blot of total protein lysates from adult oyster tissue probes stained with goat polyclonal antibodies against rat ChAT. No specific band is detected in the adult oyster tissues. The positive signal only corresponds to a protein band with a molecular weight of 69 kDa in the cell lysate of mouse spinal cord. b: Immunostaining of frozen sections of adult oyster tissues with ChAT/TUBULIN antibodies show the absence of a positive ChAT-ir signal in all tested tissues. c: Confocal image of the 7-dpf veliger stained with ChAT/TUBULIN antibodies shows no positive inner structures. The signal observed along the shell edge and in the center of the larval body is likely non-specific fluorescence. Scale bar = 100 μm in b and 50 μm in c. (TIFF 2992 kb) [file 12983_2018_259_MOESM2_ESM.tif]
